# A systematic analysis of the global disease burden of type 2 diabetes mellitus attributable to high intake of processed meat in 204 countries (1990-2021)

**DOI:** 10.3389/fendo.2025.1635831

**Published:** 2025-09-30

**Authors:** Wangchu Ze, Rui Su, Ruiyu Huang, Yanxia Guo, Xiaofang Yang, Baolu Zhang

**Affiliations:** ^1^ Department of Nursing, The Affiliated Hospital, Southwest Medical University, Luzhou, China; ^2^ School of Nursing, Southwest Medical University, Luzhou, China; ^3^ School of Continuing Education, Guiyang Healthcare Vocational University, Guiyang, China; ^4^ Faculty of Nursing and Midwifery, Jiangsu College of Nursing, Huaian, China; ^5^ Guiyang Maternal and Child Health Care Hospital, Guiyang, China

**Keywords:** global burden, type 2 diabetes mellitus, high intake of processed meat, mortality, disability-adjusted life years, socio-demographic index

## Abstract

**Background:**

High intake of processed meat is a modifiable risk factor for type 2 diabetes mellitus (T2DM), yet its global disease burden across different socio-demographic contexts remains insufficiently characterized.

**Methods:**

We analyzed T2DM mortality and disability-adjusted life years (DALYs) attributable to high processed meat intake across 204 countries from 1990 to 2021 using Global Burden of Disease 2021 data. We calculated age-standardized mortality rates (ASMR), age-standardized DALY rates (ASDR), and estimated annual percentage changes (EAPC) to assess temporal trends. Advanced interrupted time series (ITS) analysis was employed to detect structural breaks during the COVID-19 pandemic. Multivariate regression models examined the nonlinear relationship between the socio-demographic index (SDI) and the burden trend.

**Results:**

Processed meat consumption accounted for 20.3% (95% UI: 18.3-23.5) of global T2DM burden in 2021. Attributable deaths increased 118% from 52.7 to 114.9 thousand, while DALYs tripled from 2.0 to 6.1 million over three decades. Quadratic regression identified a critical SDI range (0.47-0.55) where burden growth peaks. Interrupted time series revealed pandemic-induced trend reversals: mortality declined (EAPC: +0.23% to -0.50%) while disability burden continued rising. Intervention modeling showed 10%, 25%, and 50% consumption reductions could prevent 2,330, 5,800, and 11,600 deaths annually, respectively.

**Conclusion:**

The global burden of T2DM attributable to processed meat consumption has increased substantially over three decades, with critical growth periods in middle-SDI countries. The COVID-19 pandemic disrupted established trends, revealing complex interactions between socioeconomic factors and dietary risk patterns. Targeted interventions reducing processed meat consumption could prevent thousands of deaths annually, particularly in countries within the critical SDI transition zone.

## 1 Introduction

Diabetes mellitus has emerged as a formidable global public health challenge, with its prevalence rising dramatically over the past few decades. According to the latest International Diabetes Federation (IDF) Diabetes Atlas, the global diabetes population has increased markedly from 153 million in 1980 to 537 million in 2021, with projections suggesting a continued upward trajectory reaching approximately 643 million by 2030 (1). This epidemic imposes a dual burden: at the individual level, it leads to debilitating complications including cardiovascular disease, nephropathy, and retinopathy; at the societal level, it creates substantial economic pressures through direct healthcare costs and indirect productivity losses (2, 3). In regions with limited healthcare resources, diabetes-related expenses exacerbate health inequities, while the global competition for resources further emphasizes the priority of preventive strategies (4). Type 2 diabetes mellitus (T2DM), accounting for over 90% of all diabetes cases worldwide, represents a complex metabolic disorder characterized by progressive insulin resistance and relative insulin deficiency (1, 5). Among the multiple modifiable risk factors for T2DM, dietary patterns—particularly processed meat consumption—have emerged as critical determinants (6). While processed and unprocessed meat consumption have been associated with T2DM risk, processed meat poses particularly elevated risks due to its unique compositional characteristics. A recent individual-participant federated meta-analysis of 1.97 million adults demonstrated that processed meat consumption carries a 15% higher T2DM risk per 50g daily intake (HR = 1.15, 95% CI: 1.11–1.20) compared to 10% for unprocessed red meat per 100g daily intake (HR = 1.10, 95% CI: 1.06–1.15), highlighting the distinct pathogenic potential of processed varieties (6). This differential risk profile stems from processed meat’s high content of preservatives (sodium nitrite/nitrate), advanced glycation end products (AGEs) from high-temperature processing, excess sodium, and modified protein structures—components largely absent or present in lower concentrations in fresh red meat (7, 8). Furthermore, processed meat consumption patterns tend to cluster with other unhealthy dietary behaviors and are more strongly associated with socioeconomic factors than unprocessed meat consumption, amplifying their public health significance (9).

The biological plausibility of this association is supported by multiple pathogenic pathways that exhibit important sex and age dependencies. Processed meats contain substantial amounts of preservatives such as sodium nitrite and sodium nitrate, which have been shown to induce pancreatic β-cell apoptosis and impair insulin secretion in experimental models (9, 10). The impact of these preservatives appears more pronounced in male subjects, likely due to sex-specific differences in nitric oxide metabolism (11, 12). Additionally, advanced glycation end products (AGEs), formed during high-temperature processing of meats, accumulate in tissues to promote oxidative stress and systemic inflammation, with postmenopausal women showing greater AGE-related metabolic dysregulation due to the loss of estrogen’s protective effects (13). The high content of heme iron in processed meats further contributes to T2DM risk by inducing oxidative stress and dysregulating gut microbiota composition, particularly reducing beneficial short-chain fatty acid-producing bacteria (14). This dysbiosis effect is more prominent in younger adults (20-40 years), whose gut microbiome demonstrates higher plasticity and vulnerability to dietary insults (15). Despite substantial evidence linking processed meat intake to T2DM risk, the global burden of T2DM attributable specifically to this dietary factor remains inadequately characterized. Previous Global Burden of Disease (GBD) analyses have established that dietary risks collectively account for approximately 25.7% of T2DM-related disability-adjusted life years (DALYs) globally (16), but comprehensive analyses of processed meat’s specific contribution across different regions, sociodemographic contexts, age groups, and sex are lacking. This knowledge gap is particularly concerning given recent dietary shifts, such as the COVID-19 pandemic-induced increase in processed food consumption in many regions due to supply chain disruptions and extended home confinement (17). The limited understanding of regional disparities in processed meat-related T2DM burden, especially in low- and middle-income countries experiencing rapid nutrition transitions, further hampers the development of targeted interventions and public health policies.

To address these critical gaps, our study utilizes data from the GBD 2021 to systematically quantify the burden of T2DM attributable to high intake of processed meat across 204 countries and territories from 1990 to 2021. Our specific research objectives were to: (1) characterize global T2DM burden attributable to processed meat across 204 countries from 1990-2021; (2) examine variations by sociodemographic development, age, and sex; (3) assess temporal trends including pandemic-related structural breaks using interrupted time series analysis; (4) identify drivers of burden variation through multivariate modeling; and (5) quantify intervention impact potential through scenario-based modeling. This comprehensive analysis will provide essential evidence to guide targeted prevention strategies and inform public health policy across diverse global contexts.

## 2 Method

### 2.1 Data sources and quality assurance

This study employed a comparative risk assessment (CRA) approach as implemented in the Global Burden of Disease Study 2021 (GBD 2021) to quantify the disease burden of type 2 diabetes mellitus attributable to high processed meat intake. The CRA framework estimates the proportion of disease burden that could be avoided if exposure to a risk factor were reduced to an alternative (counterfactual) exposure scenario, specifically the theoretical minimum risk exposure level (TMREL). This approach quantifies attributable burden by comparing observed exposure distributions with optimal exposure levels, utilizing established exposure-outcome relationships derived from systematic reviews and meta-analyses of epidemiological evidence. This study used data from the GBD 2021 Global Health Data Exchange (GHDX) results tool (http://ghdx.healthdata.org/gbd-results-tool). This platform collects information on incidence, prevalence, mortality, and years lived with disability for 369 diseases and injuries, as well as comparative risks for 87 risk factors across 204 countries and territories from 1990 to 2021. The detailed methodology of GBD 2021 has been reported in previous studies (18). The appropriate code was selected from GBD Compare to ensure reproducibility. As the GBD 2021 data are publicly available, this study was exempt from institutional ethics committee review (19).

The GBD 2021 framework synthesizes data from multiple established sources to ensure comprehensive global coverage. For dietary risk factors specifically, the GBD incorporates: Food and Agriculture Organization of the United Nations (FAO) Food Balance Sheets and Supply Utilization Accounts for food availability data across 195 countries; 305 dietary surveys including 24-hour dietary recalls and food frequency questionnaires; World Health Organization (WHO) Global Health Observatory mortality databases; and peer-reviewed epidemiological studies identified through systematic literature reviews. To address potential heterogeneity from differences in dietary assessment methodology across countries and periods, GBD 2021 employs the network meta-regression tool (MR-BRT) to create crosswalks between different measurement approaches. Data quality is ensured through standardized protocols, including representativeness assessment using Socio-demographic Index-based weighting and uncertainty quantification through 1,000 draw-level Bayesian posterior distributions (20).

We extracted data on T2DM deaths, total disability-adjusted life years (DALYs), crude mortality rate, crude DALY rates, age-standardized mortality rate (ASMR), and age-standardized DALY rates (ASDR) per 100,000 population attributable to high intake of processed meat, along with Socio-demographic Index (SDI) data for 204 countries and territories from 1990 to 2021. Unlike previous GBD analyses that have examined dietary risks collectively or combined red and processed meat into composite risk factors, this study specifically isolates processed meat consumption as an independent risk factor for T2DM. Additionally, this analysis uniquely extends through the COVID-19 pandemic period (2020-2021), enabling the first systematic assessment of how global health emergencies may influence diet-related chronic disease burden patterns. The study provides the most comprehensive temporal analysis to date (31 years) of processed meat-attributable T2DM burden across all development contexts. This analysis fills a critical knowledge gap in nutrition transition epidemiology and pandemic-related health system impacts.

### 2.2 Definition of type 2 diabetes mellitus and high intake of processed meat

Type 2 diabetes mellitus was defined as fasting plasma glucose (FPG) ≥126 mg/dL (7 mmol/L) or self-reported use of diabetes medication or insulin treatment, with International Classification of Diseases (ICD) version 10 codes: E11-E11.1, E11.3-E11.9 (21). However, in GBD 2021, additional indicators such as oral glucose tolerance and postprandial glucose tests were also recognized as supplementary diagnostic criteria (16).

Processed meat includes meat products that have been transformed through salting, curing, fermentation, smoking, or other processes to enhance flavor or improve preservation, such as sausages, ham, bacon, salami, and canned meat products (22). Global data on processed meat consumption were compiled from 305 sources worldwide, including national nutrition surveys, household budget surveys, and dietary recall studies (23). High intake of processed meat, categorized as a Level 2 risk factor in the GBD 2021 framework, was operationally defined as any consumption above the theoretical minimum risk exposure level (TMREL) of 0 g/day (20). This conservative threshold reflects methodological considerations in comparative risk assessment, rather than definitive evidence for the absence of safe consumption levels. Contemporary dose-response meta-analyses demonstrate approximately log-linear relationships between processed meat intake and type 2 diabetes risk across observed consumption ranges, without clear inflection points suggesting risk plateaus at lower intake levels (6). However, this parameterization acknowledges uncertainty in extrapolating epidemiological associations to very low exposure levels and potential heterogeneity in dose-response relationships across populations with different baseline risk profiles and dietary contexts.

The Socio-demographic Index (SDI) represents a composite measure of development status calculated from lagged distributed income per capita, mean years of education for individuals aged 15 years and older, and total fertility rate under age 25 (18). Following established GBD methodology, we categorized the 204 countries and territories into five SDI quintiles: high SDI (>0.805129), high-middle SDI (0.689504–0.805129), middle SDI (0.607679–0.689504), low-middle SDI (0.454743–0.607679), and low SDI (≤0.454743). Geographic analysis employed the 21 standard GBD regional classifications.

Age stratification utilized 15 categories: 14 five-year intervals spanning ages 25-94 years and one category for individuals ≥95 years. The analytical focus on adults aged ≥25 years aligns with established epidemiological frameworks recognizing this as the threshold for reliable dietary pattern assessment in population-based surveillance and the age at which type 2 diabetes mellitus incidence demonstrates sufficient population-level variation for meaningful trend analysis (24, 25).

### 2.3 Statistical analysis

We used Estimated Annual Percentage Change (EAPC) to evaluate trends in ASMR and ASDR attributable to high intake of processed meat from 1990 to 2021. EAPC is widely used to measure trends in age-standardized rate over a specific period. The calculation method employed a linear regression model as follows:


In(ASMR or ASDR)=α+βx +ϵ

EAPC= 100× (exp (β)−1)

Where x represents the calendar year, ϵ is the error term, and β describes the positive or negative trend in the age-standardized rate. EAPC and its 95% confidence interval (CI) were obtained from this model. When both the EAPC value and its 95% CI were >0, the trend was considered increasing; when both values were<0, the trend was considered decreasing; otherwise, the burden of T2DM attributable to high intake of processed meat was regarded as stable. To address limitations of traditional EAPC models in capturing non-linear temporal patterns, particularly during the COVID-19 pandemic, we conducted supplementary interrupted time series (ITS) analysis using the ‘segmented’ package in R. This approach explicitly models structural breaks in temporal trends and provides a more rigorous assessment of pandemic impacts compared to simple period-specific EAPC comparisons. The ITS models identified potential breakpoints in burden trajectories and estimated separate slope parameters for pre- and post-break periods, with 2020 specified as the primary candidate breakpoint corresponding to pandemic onset.

Given the unprecedented global disruptions caused by the COVID-19 pandemic, we analyzed temporal trends by comparing the pre-pandemic period (1990-2019) with the pandemic period (2020-2021) to assess potential pandemic-related impacts on T2DM burden trends. We calculated separate EAPC values for these two periods and compared disease burden patterns across different SDI regions before and during the pandemic. Additionally, we examined the absolute changes in ASMR and ASDR between 2019 and 2021 to identify immediate pandemic effects. This comparative approach enabled assessment of whether traditional long-term trends were maintained or disrupted during the COVID-19 period. We acknowledge that the full impact of pandemic-related dietary and healthcare changes may take years to fully manifest (17).

Following GBD methodology, we employed methodologically appropriate uncertainty measures. For deaths, DALYs, ASMR, and ASDR, we utilized 95% uncertainty intervals (UIs) derived from 1,000 draws from Bayesian posterior distributions. These UIs comprehensively capture multiple sources of uncertainty within the Bayesian hierarchical modeling framework employed by GBD, including uncertainty in exposure data, relative risk estimates, and baseline disease rates. For temporal trend analyses quantified by EAPC, we reported 95% confidence intervals (CIs), which reflect the statistical precision of regression-based estimates. This methodological distinction adheres to established reporting standards in GBD studies and appropriately differentiates between uncertainty inherent in complex Bayesian estimations versus traditional regression analyses. Uncertainty propagation follows a hierarchical cascade: primary uncertainty from 305 dietary surveys is combined with epidemiological uncertainty from meta-regression of cohort studies, baseline disease rate uncertainty from vital registration systems, and demographic uncertainty from population estimates. These component uncertainties are integrated through Monte Carlo simulation with 1,000 posterior draws, where each iteration represents a plausible combination of all input parameters. Final burden estimates represent median values across draws, with 95% uncertainty intervals reflecting the 2.5th-97.5th percentiles of this distribution.

We conducted a comprehensive evaluation of T2DM burden attributable to high processed meat intake stratified by sex, age group, geographic location, and SDI quintile. For each stratum, we estimated the number of deaths and DALYs, corresponding age-standardized rates, and percent changes between 1990 and 2021. Additionally, we analyzed the distribution characteristics of T2DM burden across SDI regions in 2021 to examine contemporary patterns of disease burden to socioeconomic development. To move beyond descriptive stratification and identify specific drivers of burden variation across contexts, we constructed multivariate regression models examining correlates of EAPC values across countries. These ecological-level models tested hypotheses regarding the relationship between socioeconomic development (SDI), geographic region, baseline burden levels, and temporal burden trajectories. Interaction effects between SDI levels and regional classifications were formally tested to identify contexts where development status differentially influences disease burden patterns. To quantify potential policy impacts, we performed intervention scenario modeling using the population attributable fraction (PAF) methodology. Population attributable fractions were calculated using standard epidemiological formulations:


PAF=[P(RR−1)]/[P(RR−1)+1]

>Where P represents exposure prevalence and RR the pooled relative risk estimate. We evaluated three reduction scenarios based on empirical evidence: 10% reduction reflecting achievable short-term targets from food labeling policies, 25% reduction corresponding to medium-term effects from taxation policies, and 50% reduction representing the theoretical maximum feasible change based on successful public health interventions (26, 27). Preventable burden was calculated assuming linear dose-response relationships within intervention ranges, using proportional reduction methods applied to current GBD burden estimates. All scenario analyses were stratified by SDI region and incorporated existing GBD uncertainty quantification frameworks.

All statistical analyses and visualizations were performed using R software version 4.4.2. The appropriate GBD Compare codes were selected and documented to ensure reproducibility. Complete R analysis code and methodological protocols are available from the corresponding author upon reasonable request to ensure full reproducibility. The study protocol adhered to STROBE (Strengthening the Reporting of Observational Studies in Epidemiology) guidelines for reporting observational research.

## 3 Results

### 3.1 Type 2 diabetes mellitus deaths and ASMR associated with high intake of processed meat

**Figure 1:**
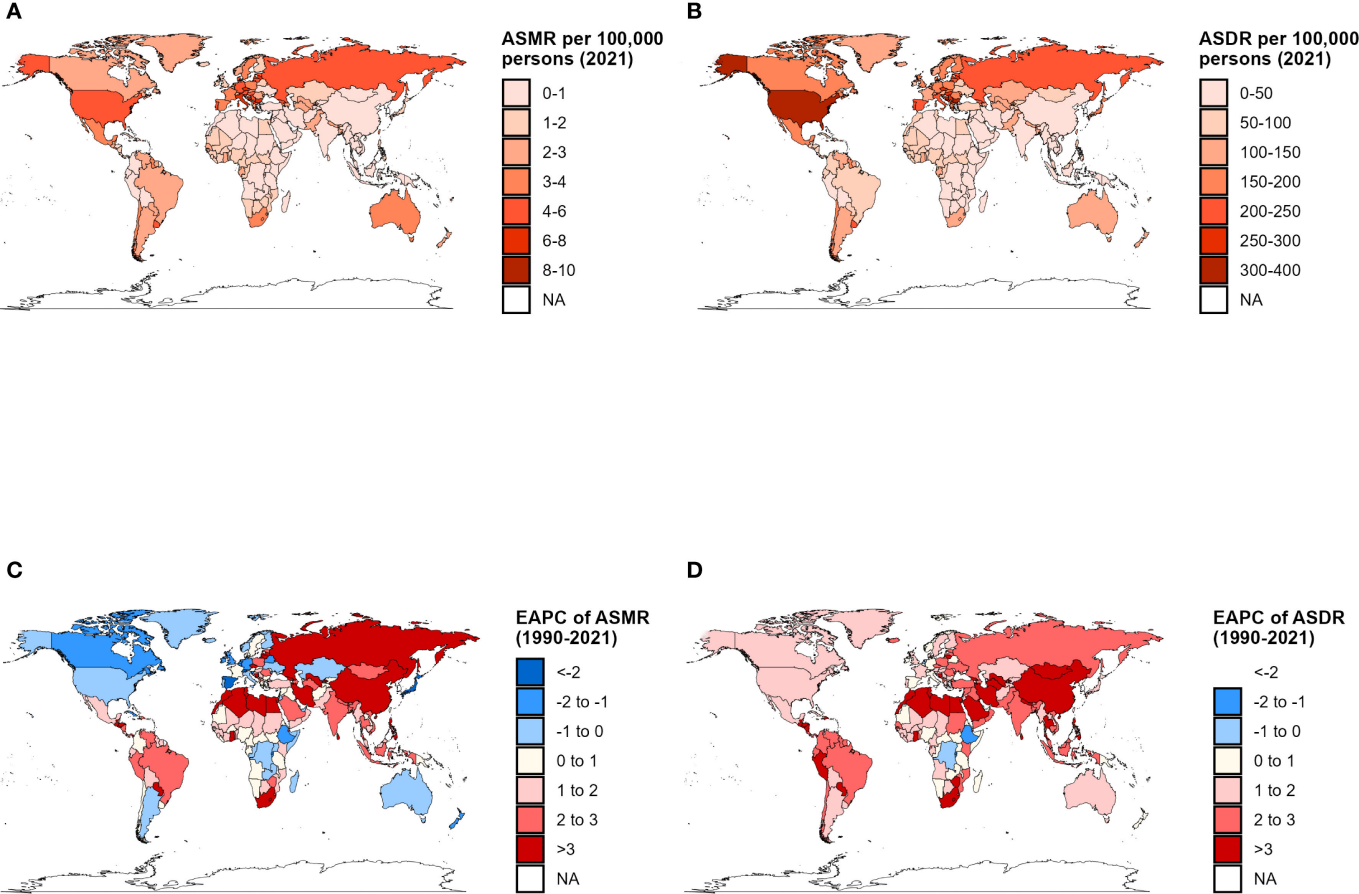
Global distribution of disease burden attributable to high intake of processed meat. **(A)** Age-standardized mortality rate (ASMR) per 100,000 persons in 2021. **(B)** Age-standardized DALY rate (ASDR) per 100,000 persons in 2021. **(C)** Percentage change in death cases from 1990 to 2021. **(D)** Percentage change in DALYs from 1990 to 2021.

Global deaths from type 2 diabetes mellitus attributable to high processed meat intake escalated dramatically from 52.7 thousand (95% UI: 12.5–85.9) in 1990 to 114.9 thousand (95% UI: 26.8–190.9) in 2021, representing a 118% increase (**Table 1**). Despite this substantial surge in absolute numbers, the global ASMR demonstrated a modest decline from 1.5 (95% UI: 0.4–2.5) to 1.4 (95% UI: 0.3–2.3) per 100,000, with an EAPC of −0.46 (95% CI: −0.56 to −0.35), indicating a consistent downward trajectory in mortality rates over the 31 years (**Figure 1**). Regional patterns revealed striking disparities across different SDI levels. High SDI regions experienced a notable reduction in ASMR from 2.2 (95% UI: 0.5–3.6) to 1.5 (95% UI: 0.4–2.5) per 100,000, with an EAPC of −1.56 (95% CI: −1.82 to −1.29), reflecting pronounced improvements. Conversely, middle and low-middle SDI regions exhibited opposing upward trajectories, with EAPCs of 1.13 (95% CI: 1.05–1.21) and 1.46 (95% CI: 1.39–1.52), respectively. Low SDI regions maintained relatively stable patterns, with ASMR climbing marginally from 2.0 (95% UI: 0.5–3.4) to 2.2 (95% UI: 0.5–3.8) and an EAPC of 0.32 (95% CI: 0.27–0.36).

**Table 1:** Deaths and ASMR of T2DM attributable to high intake of processed meat.

Characteristic	Death cases, n × 10^3^ (95% UI) (1990)	ASMR per 10^5^, n (95% UI) (2021)	Death cases, n × 10^3^ (95% UI) (2021)	ASMR per 10^5^, n (95% UI) (2021)	EAPC ASMR, n (95% CI) (1990-2021)
Global	52.7 (12.5-85.9)	1.5 (0.4-2.5)	114.9 (26.8-190.9)	1.4 (0.3-2.3)	-0.46 (-0.56–0.35)
SDI					
High SDI	24.6 (5.9-39.8)	2.2 (0.5-3.6)	34.6 (8.3-56.3)	1.5 (0.4-2.5)	-1.56 (-1.82–1.29)
High-middle SDI	12.2 (2.9-19.7)	1.4 (0.3-2.2)	24.9 (5.7-40.7)	1.3 (0.3-2.1)	-0.36 (-0.55–0.16)
Middle SDI	6.1 (1.4-10.3)	0.7 (0.2-1.1)	23.5 (5.4-40.4)	0.9 (0.2-1.6)	1.13 (1.05-1.21)
Low-middle SDI	5.6 (1.3-9.4)	1.1 (0.3-1.8)	21.8 (5.0-36.5)	1.7 (0.4-2.8)	1.46 (1.39-1.52)
Low SDI	4.1 (1.0-6.9)	2.0 (0.5-3.4)	10.1 (2.3-17.0)	2.2 (0.5-3.8)	0.32 (0.27-0.36)
Region					
Andean Latin America	0.1 (0.0-0.2)	0.4 (0.1-0.8)	0.4 (0.1-0.6)	0.6 (0.1-1.1)	1.13 (0.93-1.33)
Australasia	0.5 (0.1-0.8)	2.0 (0.5-3.3)	0.9 (0.2-1.5)	1.6 (0.4-2.6)	-1.14 (-1.43–0.84)
Caribbean	0.5 (0.1-0.8)	1.8 (0.4-3.1)	0.8 (0.2-1.5)	1.6 (0.4-2.7)	-0.51 (-0.61–0.41)
Central Asia	0.6 (0.1-1.0)	1.3 (0.3-2.1)	2.0 (0.5-3.5)	2.5 (0.6-4.2)	1.83 (1.46-2.19)
Central Europe	2.5 (0.6-4.0)	1.7 (0.4-2.7)	4.7 (1.1-7.7)	2.0 (0.5-3.3)	1.02 (0.82-1.22)
Central Latin America	2.1 (0.5-3.5)	2.6 (0.6-4.3)	7.0 (1.7-12.3)	2.8 (0.7-4.9)	0.13 (-0.12-0.38)
Central Sub-Saharan Africa	0.6 (0.2-1.1)	3.1 (0.7-5.6)	1.4 (0.3-2.5)	2.9 (0.6-5.0)	-0.25 (-0.43–0.08)
East Asia	1.8 (0.4-3.1)	0.2 (0.1-0.4)	8.1 (1.8-14.3)	0.4 (0.1-0.7)	1.76 (1.54-1.99)
Eastern Europe	2.1 (0.5-3.4)	0.7 (0.2-1.2)	8.6 (2.0-13.9)	2.4 (0.6-3.8)	2.69 (1.25-4.14)
Eastern Sub-Saharan Africa	1.7 (0.4-3.0)	2.5 (0.6-4.3)	3.7 (0.8-6.2)	2.4 (0.5-4.1)	-0.24 (-0.29–0.19)
High-income Asia Pacific	2.5 (0.6-4.1)	1.2 (0.3-2.1)	2.6 (0.6-4.4)	0.5 (0.1-0.9)	-2.91 (-3.16–2.66)
High-income North America	10.2 (2.4-16.4)	2.8 (0.7-4.6)	16.2 (4.0-26.0)	2.4 (0.6-3.9)	-1.22 (-1.72–0.73)
North Africa and the Middle East	1.0 (0.2-1.7)	0.7 (0.1-1.2)	4.3 (1.0-7.3)	1.0 (0.2-1.8)	2.07 (1.81-2.34)
Oceania	0.1 (0.0-0.1)	2.5 (0.6-4.4)	0.2 (0.0-0.4)	2.8 (0.6-5.0)	0.42 (0.34-0.51)
South Asia	4.0 (0.9-6.8)	0.9 (0.2-1.5)	15.3 (3.5-26.1)	1.2 (0.3-2.1)	0.84 (0.72-0.96)
Southeast Asia	1.1 (0.3-2.0)	0.5 (0.1-0.8)	4.8 (1.1-8.1)	0.8 (0.2-1.3)	1.76 (1.70-1.81)
Southern Latin America	1.2 (0.3-2.0)	2.7 (0.6-4.5)	2.1 (0.5-3.4)	2.3 (0.6-3.8)	-0.53 (-0.73–0.33)
Southern Sub-Saharan Africa	0.5 (0.1-0.9)	2.0 (0.5-3.4)	2.3 (0.5-3.9)	4.3 (1.0-7.4)	3.11 (2.62-3.61)
Tropical Latin America	1.3 (0.3-2.3)	1.5 (0.3-2.6)	4.7 (1.1-8.1)	1.9 (0.4-3.2)	1.03 (0.84-1.23)
Western Europe	16.0 (3.8-26.0)	2.7 (0.6-4.3)	17.5 (4.1-28.4)	1.5 (0.4-2.5)	-1.81 (-1.93–1.69)
Western Sub-Saharan Africa	2.4 (0.6-3.9)	3.0 (0.7-5.0)	7.3 (1.8-12.2)	4.2 (1.1-7.1)	1.16 (1.01-1.31)

ASMR, Age-Standardized Mortality Rate; EAPC, Estimated Annual Percentage Change; UI, Uncertainty Interval; CI, Confidence Interval; SDI, Socio-demographic index. *EAPC value and its 95% CI > 0 indicates an increasing trend. † EAPC value and its 95% CI< 0 indicates a decreasing trend.

Among GBD regions, Eastern Europe demonstrated the most dramatic increase, with ASMR rising from 0.7 (95% UI: 0.2–1.2) to 2.4 (95% UI: 0.6–3.8) per 100,000, with an EAPC of 2.69 (95% CI: 1.25–4.14). Southern Sub-Saharan Africa also witnessed remarkable growth with an EAPC of 3.11 (95% CI: 2.62–3.61). In stark contrast, High-income Asia Pacific recorded substantial decreases in ASMR with an EAPC of −2.91 (95% CI: −3.16 to −2.66), while Western Europe experienced considerable downward trends with an EAPC of −1.81 (95% CI: −1.93 to −1.69). At the national level, Bosnia and Herzegovina registered the highest ASMR of 9.46 (95% UI: 2.15–16.49) per 100,000 in 2021, while Morocco exhibited the most pronounced growth trajectory with an EAPC of 5.47 (95% CI: 4.76–6.19). Multiple countries in the Middle East and North Africa region, including Egypt and Morocco, displayed markedly increasing ASMR trends.

### 3.2 Global burden of type 2 diabetes mellitus associated with high intake of processed meat by sex and age

**Figure 2:**
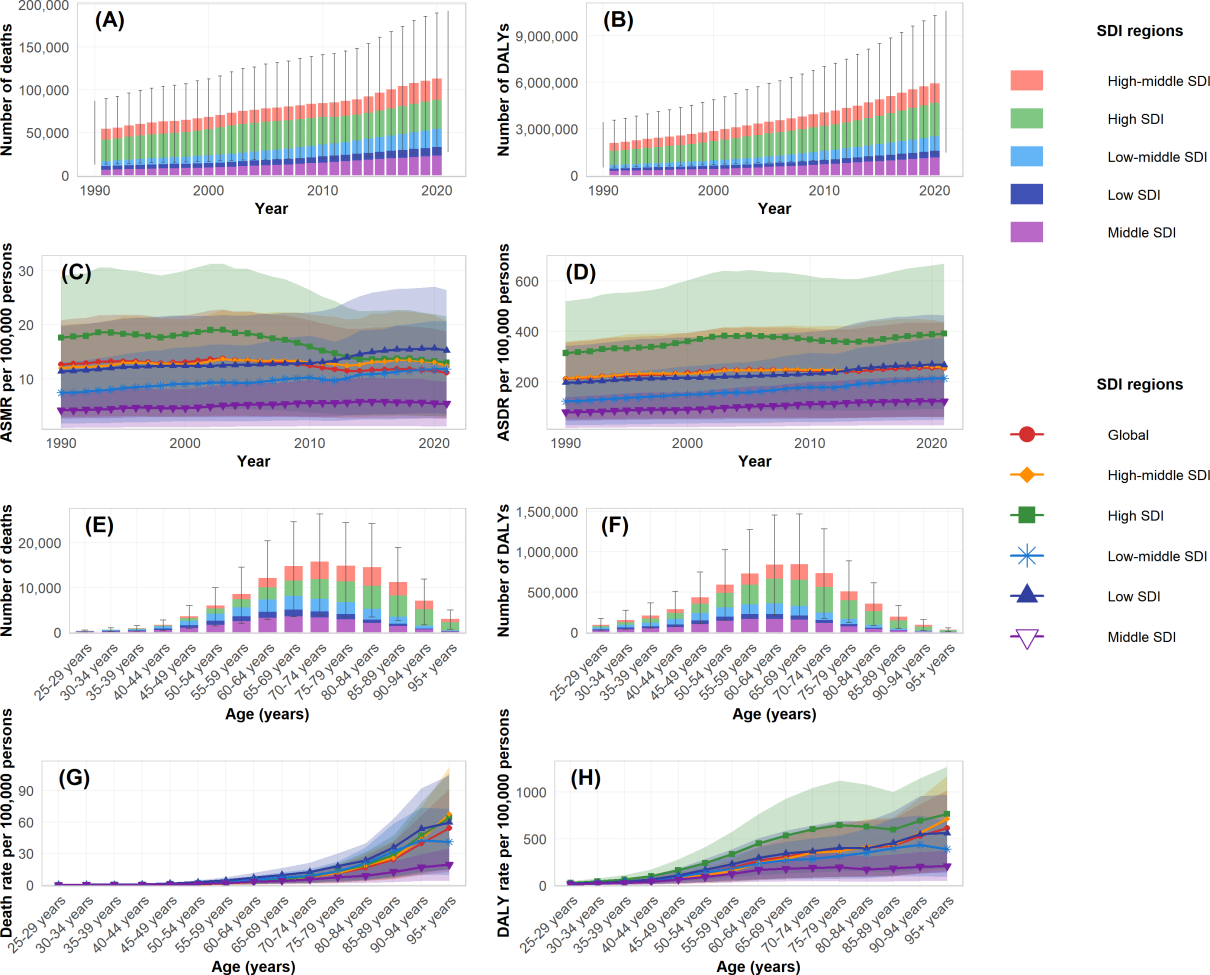
Temporal and age-specific trends of disease burden attributable to high intake of processed meat by SDI regions during 1990-2021. **(A)** Number of deaths by SDI regions. **(B)** Number of DALYs by SDI regions. **(C)** Age-standardized mortality rate (ASMR) per 100,000 persons. **(D)** Age-standardized DALY rate (ASDR) per 100,000 persons. **(E)** Number of deaths by age groups in 2021. **(F)** Number of DALYs by age groups in 2021. **(G)** Age-specific death rate per 100,000 persons in 2021. **(H)** Age-specific DALY rate per 100,000 persons in 2021.

**Figure 3:**
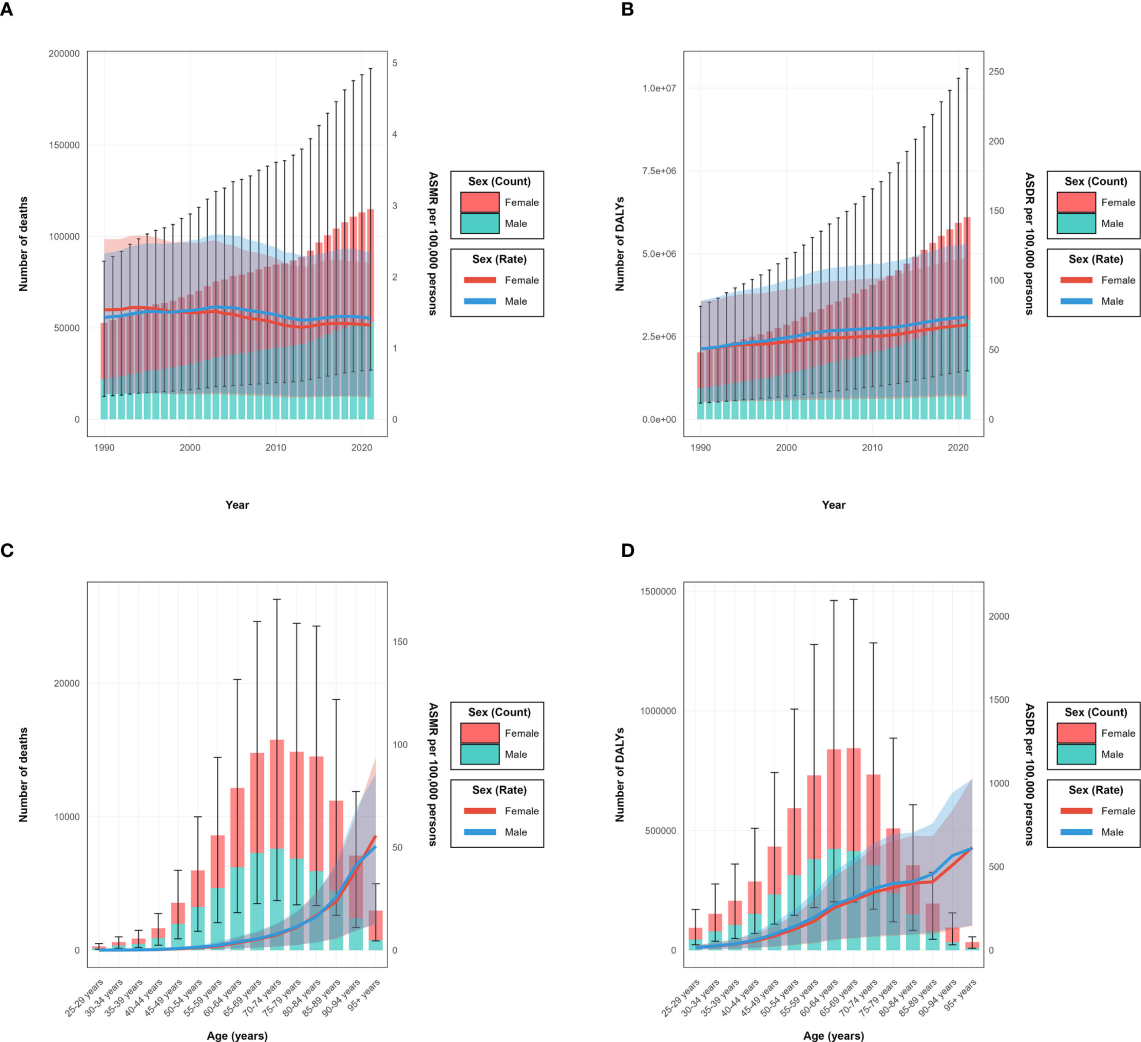
Year-specific and age-specific disease burden attributable to high intake of processed meat by sex from 1990 to 2021. **(A)** Annual number of deaths (bars, left y-axis) and age-standardized mortality rate (ASMR) per 100,000 persons (lines, right y-axis). **(B)** Annual number of disability-adjusted life years (DALYs) (bars, left y-axis) and age-standardized DALY rates (ASDR) per 100,000 persons (lines, right y-axis). **(C)** Age-specific number of deaths (bars, left y-axis) and age-specific mortality rate per 100,000 persons (lines, right y-axis) in 2021. **(D)** Age-specific number of DALYs.

The burden of type 2 diabetes mellitus attributable to high intake of processed meat in 2021 revealed distinctive patterns across demographic groups. The age-specific rate for both deaths and DALYs demonstrated a progressive increase with advancing age, with clear patterns visible across all SDI regions (**Figures 2G, H**). In absolute numbers, deaths reached their peak in the 70–74 age group with approximately 15 thousand deaths globally, while DALYs achieved their maximum in the 65–69 age group at around 800 thousand DALYs (**Figures 2E, F**). When stratified by sex, the total burden was considerably higher in females, accounting for 71.2 thousand deaths (95% UI: 37.0–115.6) and 2.51 million DALYs (95% UI: 1.25–4.30), compared to 54.0 thousand deaths (95% UI: 25.7–91.5) and 2.04 million DALYs (95% UI: 0.90–3.70) in males (**Table 2**). These patterns are demonstrated in the annual trends and sex-specific burden analysis (**Figures 3A, B**). However, the age-standardized rate showed minimal differences between sexes, with ASMR at approximately 1.6 per 100,000 for both males and females in 2021. For ASDR, females maintained marginally higher values (57.5 per 100,000, 95% UI: 28.6–98.4) than males (54.3 per 100,000, 95% UI: 25.0–97.6).

**Table 2:** DALYs and ASDR of T2D attributable to high Intake of processed meat.

Characteristic	DALYs, n × 10^3^ (95% UI) 1990	ASDR per 10^5^, n (95% UI) 1990	DALYs, n × 10^3^ (95% UI) 2021	ASDR per 10^5^, n (95% UI) 2021	EAPC ASDR, n (95% CI) (1990-2021)
Global	2018.9 (495.9-3390.3)	50.8 (12.4-85.4)	6102.9 (1479.2-10437.6)	70.6 (17.1-120.7)	1.01 (0.97-1.06)
High SDI	887.0 (219.7-1486.2)	81.6 (20.3-136.5)	2238.2 (549.6-3828.0)	119.6 (29.7-205.7)	1.08 (0.96-1.20)
High-middle SDI	501.8 (122.9-842.1)	50.6 (12.4-84.9)	1264.2 (305.5-2185.2)	65.5 (15.9-113.0)	0.79 (0.70-0.88)
Middle SDI	266.4 (65.0-462.7)	24.0 (5.8-41.9)	1174.8 (280.0-2068.6)	42.1 (10.0-74.3)	1.95 (1.88-2.02)
Low-middle SDI	213.0 (51.2-364.7)	33.4 (8.0-57.1)	974.6 (232.8-1672.4)	64.0 (15.3-110.1)	2.20 (2.16-2.24)
Low SDI	147.6 (36.3-249.5)	60.6 (14.9-102.1)	444.2 (104.9-754.1)	78.5 (18.4-134.3)	0.79 (0.76-0.82)
Region					
Andean Latin America	3.8 (0.9-6.7)	16.8 (3.8-29.7)	19.2 (4.3-33.4)	31.2 (6.9-54.4)	2.04 (1.90-2.18)
Australasia	15.9 (3.8-26.6)	68.3 (16.3-114.1)	42.4 (10.2-74.5)	83.9 (20.5-147.1)	0.50 (0.42-0.59)
Caribbean	17.1 (4.1-29.7)	64.6 (15.3-111.9)	43.5 (10.4-77.0)	81.4 (19.4-144.0)	0.76 (0.69-0.83)
Central Asia	31.2 (7.7-53.3)	63.1 (15.5-108.3)	119.9 (28.7-205.8)	132.8 (31.7-229.7)	2.28 (2.06-2.51)
Central Europe	116.1 (28.1-199.8)	77.0 (18.6-131.8)	241.1 (58.8-420.7)	115.4 (28.2-200.9)	1.60 (1.48-1.72)
Central Latin America	86.2 (20.8-148.5)	93.8 (22.6-161.3)	310.1 (77.2-548.9)	119.4 (29.6-211.7)	0.62 (0.44-0.80)
Central Sub-Saharan Africa	22.6 (5.7-39.6)	91.1 (22.6-160.0)	64.2 (14.9-111.6)	99.1 (22.5-172.8)	0.27 (0.10-0.44)
East Asia	102.8 (23.3-182.4)	11.0 (2.5-19.5)	623.5 (146.1-1123.0)	29.6 (7.0-53.5)	3.65 (3.54-3.76)
Eastern Europe	140.6 (34.9-234.3)	50.2 (12.5-83.8)	369.5 (89.9-612.7)	107.7 (26.1-179.1)	2.02 (1.62-2.42)
Eastern Sub-Saharan Africa	57.2 (14.0-98.5)	70.1 (17.1-120.4)	138.6 (32.3-235.6)	72.6 (16.6-123.5)	0.00 (-0.05-0.06)
High-income Asia Pacific	151.4 (37.7-258.1)	73.4 (18.3-125.1)	359.9 (85.9-653.7)	101.2 (24.6-180.5)	0.94 (0.78-1.09)
High-income North America	374.1 (92.7-610.8)	110.5 (27.5-180.7)	1120.8 (278.8-1903.6)	184.7 (46.5-313.2)	1.39 (1.17-1.61)
North Africa and Middle East	41.6 (9.9-72.7)	23.3 (5.5-40.8)	261.5 (61.6-462.6)	51.9 (12.1-91.0)	3.05 (2.85-3.24)
Oceania	2.5 (0.6-4.6)	74.2 (16.8-133.0)	8.6 (1.9-15.1)	97.8 (21.6-172.3)	0.86 (0.78-0.94)
South Asia	154.0 (37.1-266.6)	26.1 (6.2-45.2)	680.8 (160.6-1182.6)	44.2 (10.5-76.9)	1.64 (1.59-1.69)
Southeast Asia	43.3 (10.2-75.7)	15.8 (3.7-27.4)	205.8 (46.5-358.7)	29.7 (6.7-52.0)	2.22 (2.15-2.29)
Southern Latin America	41.8 (10.0-70.9)	89.7 (21.5-151.9)	107.7 (25.5-187.8)	126.3 (30.0-219.8)	1.08 (0.99-1.17)
Southern Sub-Saharan Africa	17.2 (4.1-29.5)	59.9 (14.1-102.2)	78.7 (18.7-134.9)	129.9 (30.6-224.5)	3.10 (2.71-3.50)
Tropical Latin America	55.0 (12.7-94.8)	55.5 (12.8-95.9)	221.4 (51.2-393.5)	84.4 (19.5-150.1)	1.71 (1.54-1.87)
Western Europe	460.3 (113.5-765.7)	81.3 (20.2-134.9)	777.0 (187.3-1328.9)	95.4 (23.3-163.4)	0.42 (0.35-0.49)
Western Sub-Saharan Africa	84.1 (20.4-139.6)	90.6 (22.0-150.3)	308.7 (74.9-523.7)	141.3 (34.2-240.3)	1.48 (1.36-1.61)

ASDR, Age-Standardized Disability Rate; EAPC, Estimated Annual Percentage Change; UI, Uncertainty Interval; CI, Confidence Interval; SDI, Socio-demographic index. * EAPC value and its 95% CI > 0 indicates an increasing trend. † EAPC value and its 95% CI< 0 indicates a decreasing trend.

The age distribution of burden varied by sex, with the peak number of deaths occurring in the 75–79 age group for females and the 70–74 age group for males. Similarly, the peak for DALYs was observed in the 70–74 age group for females and 65–69 for males, as clearly shown in the age-specific burden patterns (**Figures 3C, D**). Before age 60, burden measures were comparable between sexes, whereas after age 60, females exhibited higher absolute numbers and rates. Across SDI regions, high SDI areas showed peak burden at older ages (75–79 years) compared to middle and low-middle SDI regions, where peaks occurred at younger ages. These patterns reflect the temporal and regional distribution characteristics (**Figures 2A, B**). The ASMR and ASDR values also varied considerably by SDI region, with high SDI regions showing a declining trajectory in ASMR but increasing ASDR over the study period (**Figures 2C, D**).

### 3.3 Type 2 diabetes mellitus burden attributable to high intake of processed meat by regions and SDI

**Figure 4:**
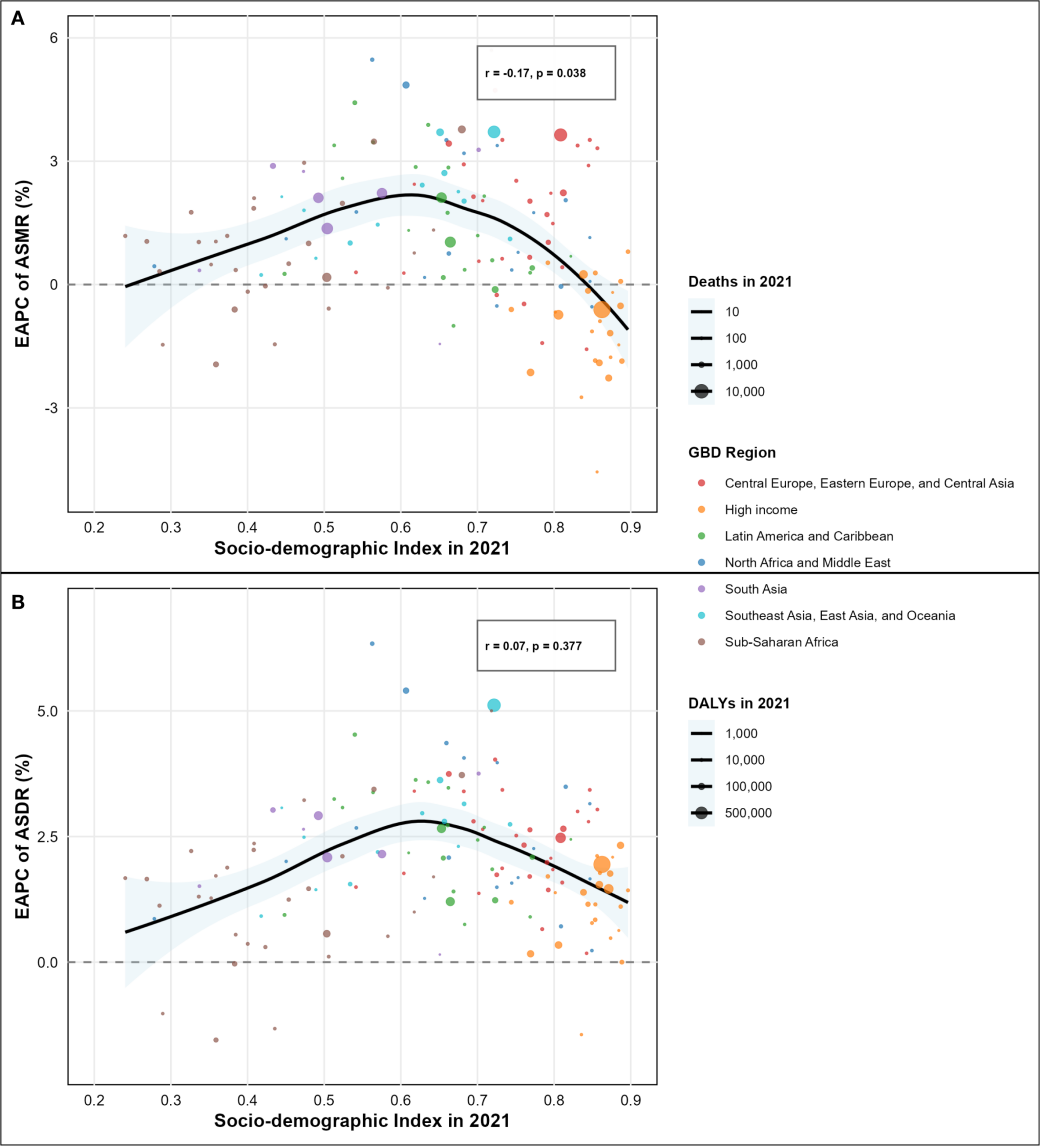
Association between socio-demographic index (SDI) and estimated annual percentage change (EAPC) of disease burden attributable to high intake of processed meat by country. **(A)** Correlation between SDI and EAPC of age-standardized mortality rate (ASMR) from 1990 to 2021 (r = -0.19, p = 0.007). **(B)** Correlation between SDI and EAPC of age-standardized disability-adjusted life years (ASDR) from 1990 to 2021 (r = 0.05, p = 0.441).

**Figure 5:**
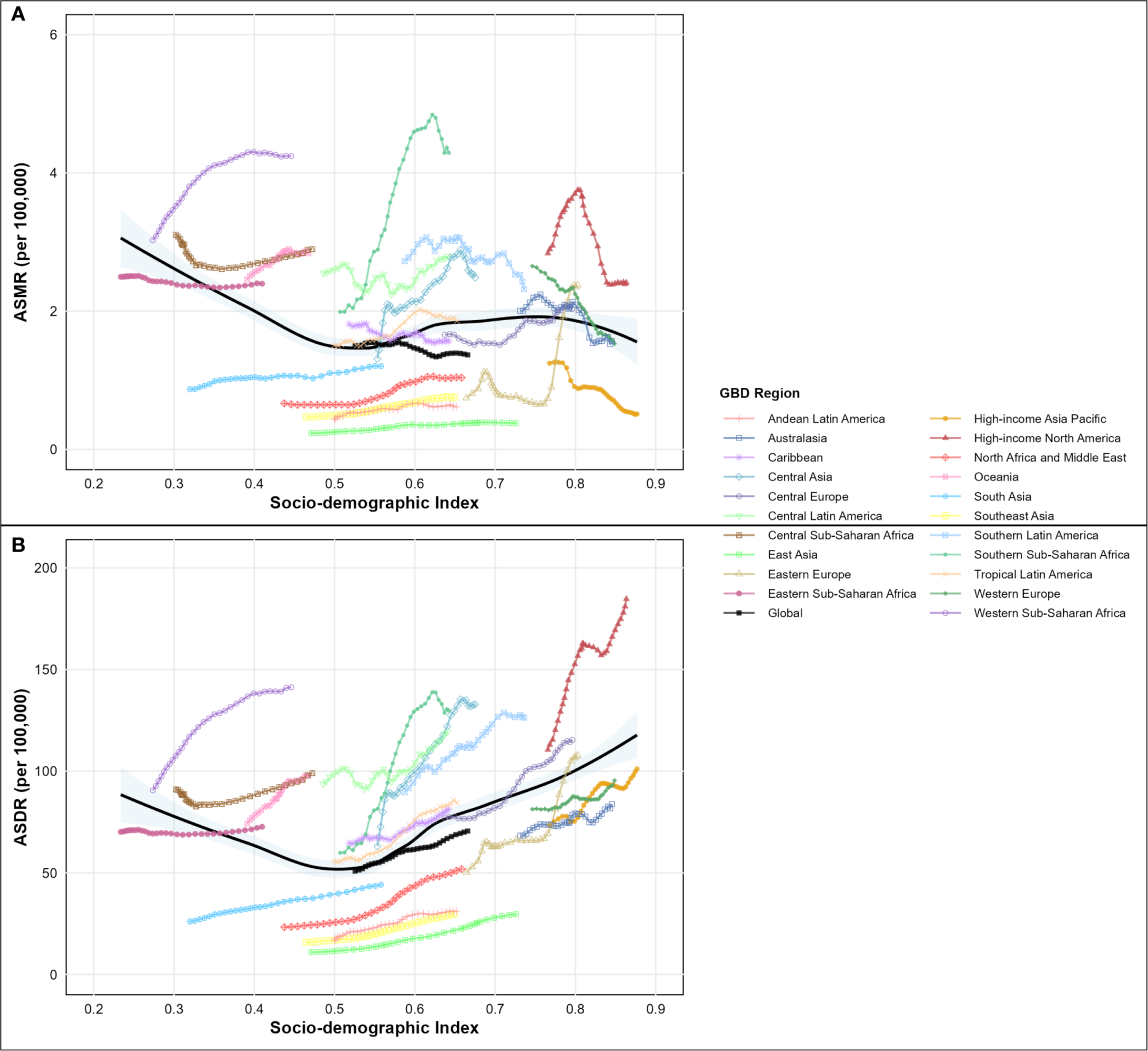
Age-standardized rate of disease burden attributable to high intake of processed meat across the socio-demographic index (SDI) spectrum in 2021, by global regions. **(A)** Age-standardized mortality rate (ASMR) per 100,000 people by SDI level. **(B)** Age-standardized disability-adjusted life year (DALY) rate per 100,000 people by SDI level.

Analysis of type 2 diabetes mellitus burden attributable to high intake of processed meat from 1990-2021 unveiled substantial regional variations across different GBD regions and socio-demographic index levels, as comprehensively illustrated in the SDI-burden relationship analysis (**Figures 4A, B**; **Figures 5A, B**). Southern Sub-Saharan Africa and Andean Latin America regions exhibited the highest ASMR, with Oceania showing peak values at higher SDI levels and Southern Sub-Saharan Africa exceeding 4.5 per 100,000 at SDI values around 0.6, clearly visible in the regional burden distribution across the SDI spectrum (**Figure 5A**). The temporal trends reflected in EAPC values show Morocco and Mauritius experiencing the most significant escalations in ASMR, contrasting sharply with high-income countries that generally demonstrated declining trajectories, with Singapore showing the steepest decrease. These patterns yielded a negative correlation between SDI and EAPC of ASMR (r = -0.17, p = 0.038), suggesting that more developed regions typically experienced greater reductions in mortality over time, as demonstrated in the correlation analysis (**Figure 4A**). For disability-adjusted life years, Oceania, Andean Latin America, and Southern Sub-Saharan Africa carried the heaviest burdens, with ASDR values reaching 180, 140, and 140 per 100,000 population, as shown in the regional DALY burden distribution (**Figure 5B**). While the global ASDR pattern showed initial decreases with rising SDI (0.2-0.5) followed by increases at higher development levels, regional trajectories varied considerably. The EAPC analysis for ASDR revealed Morocco, Egypt, and Bosnia and Herzegovina experiencing the most substantial escalations, while the correlation between SDI and EAPC of ASDR was weak and not statistically significant (r = 0.07, p = 0.377), as illustrated in the SDI-EAPC relationship (**Figure 4B**). This contrasts with ASMR trends, highlighting that while mortality improvements were associated with development, changes in overall disease burden (including non-fatal outcomes) followed more complex patterns not linked to socio-demographic development alone.

Advanced statistical modeling revealed that the relationship between socio-demographic development and disease burden trends follows a nonlinear quadratic pattern rather than the simple linear associations suggested by correlation analysis. Multivariate quadratic regression, controlling for baseline disease burden, demonstrated that the relationship between SDI and EAPC follows a significant inverted-U pattern for mortality trends (R² = 0.357, p< 0.001 for quadratic term), substantially outperforming simple linear models (R² = 0.264). The quadratic function identified an optimal SDI point of 0.468, where mortality burden growth reaches its peak, creating a relationship where countries at middle-income development levels experienced the highest EAPC values while both lower and higher SDI countries showed relatively lower burden growth rates (**Supplementary Figure S1**). For disability burden trends, a similar but less pronounced quadratic relationship emerged (R² = 0.296, p = 0.001 for quadratic term), with the optimal SDI point at 0.549, indicating that the middle-income development phase represents the critical period for diabetes burden escalation attributable to processed meat consumption (**Supplementary Figure S1**). Validation using the GBD standard SDI quintile classification confirmed the quadratic relationship through systematic group differences. Low-middle SDI countries (SDI 0.455-0.608, n=52) demonstrated the highest mean EAPC of 1.47 ± 1.24% per year, followed by middle SDI countries (SDI 0.608-0.690, n=31) with 1.02 ± 1.18% per year, while high SDI countries (SDI >0.805, n=45) showed negative mean EAPC of -0.58 ± 1.52% per year (F = 18.7, p< 0.001, one-way ANOVA) (**Supplementary Figure S2**). *Post-hoc* analysis confirmed significant differences between high and low-middle SDI groups (difference = -2.05% per year, 95% CI: -2.74 to -1.36, p< 0.001), validating the quadratic model’s identification of middle-income countries as the highest-risk development phase. The critical SDI range of 0.47-0.55 emerged as the highest-risk development phase, where countries experience rapid dietary transition toward processed food consumption, insufficient regulatory frameworks for food policy, healthcare systems unprepared for non-communicable disease burden escalation, and limited public health infrastructure for prevention programs.

Both absolute burden and change rate analyses illustrate important geographic variations. Regions like Central Asia and Central Latin America showed increasing disease burden with rising SDI, while High-income Asia Pacific demonstrated decreasing burden despite high development. East Asia, South Asia, and North Africa maintained a relatively lower burden throughout the study period. Countries in the critical middle-income range, including many in Central Asia, Eastern Europe, and North Africa, showed the steepest burden increases, with Morocco demonstrating the most extreme escalation (EAPC of 6.34% for ASDR and 5.47% for ASMR). These diverse regional patterns highlight the complex interplay between processed meat consumption, socioeconomic development, and health system capacities in determining both the magnitude and trajectory of type 2 diabetes mellitus burden attributable to dietary factors across different global contexts. The nonlinear relationship demonstrates that the nutrition transition period represents a window of maximum vulnerability, where targeted interventions could prevent the escalation of diet-related diabetes burden more effectively than interventions applied uniformly across all development levels.

### 3.4 Impact of COVID-19 pandemic on type 2 diabetes mellitus burden related to high intake of processed meat

The COVID-19 pandemic appeared to influence the trajectory of the T2DM burden attributable to high processed meat intake, though individual coefficient estimates did not achieve statistical significance. Differential impacts were observed across multiple analytical approaches and sociodemographic strata.

**Figure 6:**
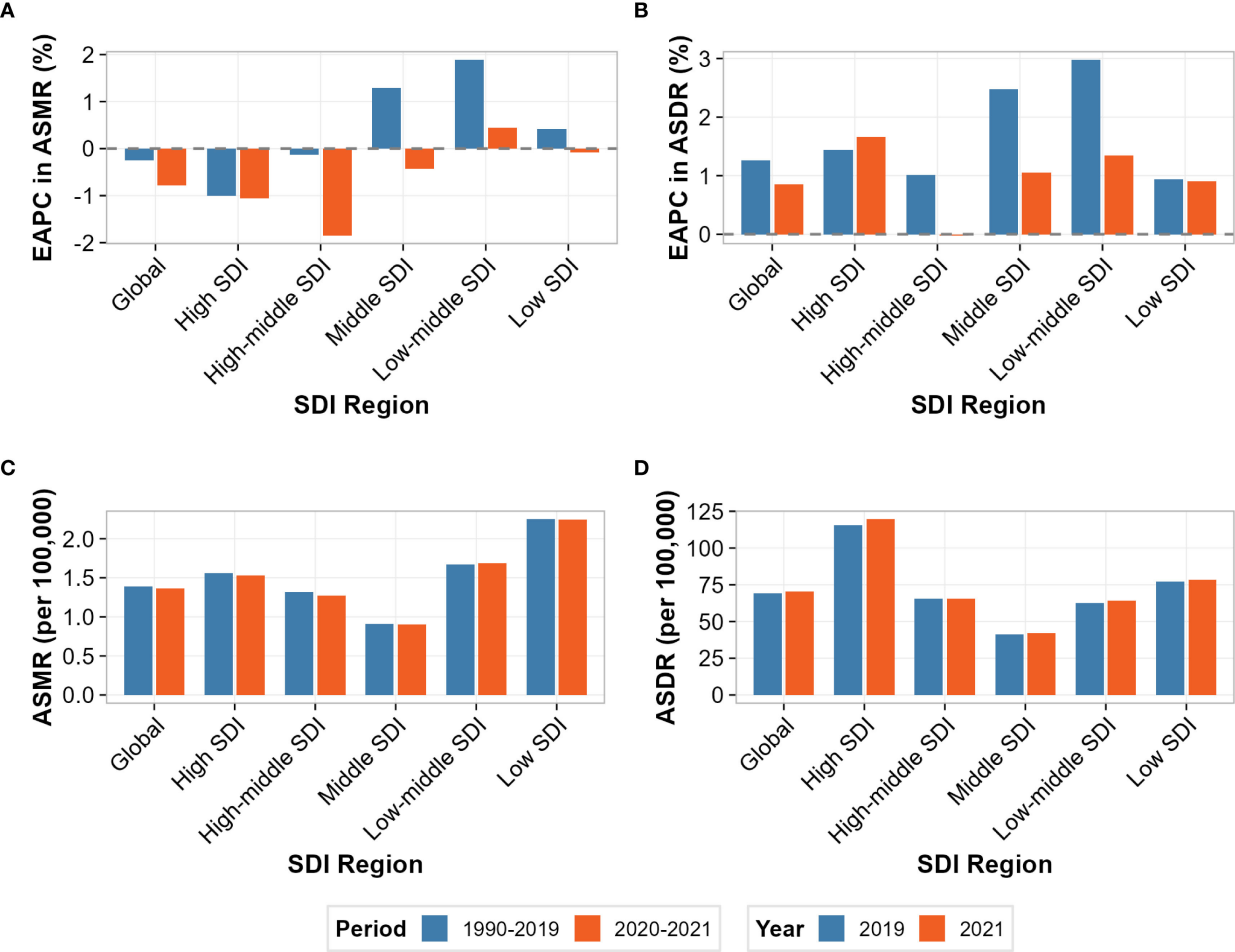
Comparison of disease burden attributable to high intake of processed meat before and during the COVID-19 pandemic across socio-demographic index (SDI) regions. **(A)** Estimated annual percentage change (EAPC) in age-standardized mortality rate (ASMR) before COVID-19 (1990-2019) and during COVID-19 (2020-2021). **(B)** Estimated annual percentage change (EAPC) in age-standardized disability-adjusted life year rate (ASDR) before and during COVID-19. **(C)** ASMR values (per 100,000 persons) in 2019 (pre-COVID) and 2021 (during COVID). **(D)** ASDR values (per 100,000 persons) in 2019 and 2021.

Temporal trend analysis comparing pre-pandemic (1990-2019) and pandemic (2020-2021) periods revealed heterogeneous modifications in disease burden trajectories (**Figure 6**). For age-standardized mortality rates (**Figure 6A**), divergent patterns emerged across SDI regions, with low-middle SDI demonstrating the most pronounced trend reversal, shifting from positive pre-pandemic growth to negative trajectories during 2020-2021. High SDI regions maintained declining trends throughout both periods, though with modified slopes. For age-standardized disability rates (**Figure 6B**), all regions sustained positive growth trajectories during both epochs, but with varying magnitude changes—high SDI regions showed acceleration while low-middle SDI regions exhibited deceleration. ASMR demonstrated relative stability across most regions, with changes ranging from -2.1% in high SDI to +8.3% in low SDI regions (**Figure 6C**). The largest absolute increase occurred in low SDI regions (from 2.04 to 2.21 per 100,000), while high SDI regions showed minimal change (from 1.54 to 1.51 per 100,000). In contrast, ASDR exhibited consistent increases across all SDI strata, with high SDI regions experiencing the most substantial relative increase of 5.7% (from 113.2 to 119.6 per 100,000), followed by low-middle SDI with a 3.2% increase (**Figure 6D**).

Heat map visualization of ITS results suggested potential regional patterns, though statistical significance was not achieved (all p>0.05) (**Supplementary Figure S3**). For immediate effects (2020 level changes), all regions showed positive ASDR changes, with high-middle SDI demonstrating the largest magnitude (+2.442 per 100,000), followed by middle SDI (+1.100) and low SDI (+1.009). ASMR immediate effects showed greater heterogeneity, with high-middle SDI showing the largest positive change (+0.120 per 100,000), while high SDI (-0.036) and middle SDI (-0.012) exhibited negative changes. For trend modifications (2020-2021 slope changes), most regions demonstrated negative values for both outcomes, suggesting deceleration of pre-pandemic trajectories, with the notable exception of high SDI showing positive ASDR trend change (+1.289 per 100,000/year). Time series visualization confirmed these structural breaks, with 2020-2021 data points (triangular markers) systematically deviating from pre-pandemic linear trends established during 1990-2019 (**Supplementary Figure S4**). The divergence was most pronounced for ASDR in high and high-middle SDI regions, while ASMR showed more subtle modifications across all strata. Correlation analysis of immediate pandemic effects across SDI regions revealed a strong positive association between mortality and disability responses (**Supplementary Figure S5**). The Pearson correlation coefficient of r = 0.920 indicates that regions experiencing larger immediate ASMR changes generally also experienced proportional ASDR changes, suggesting synchronized pandemic impacts across health outcomes. In the quadrant analysis, most regions clustered in the upper-right quadrant (both ASMR and ASDR increases), with high-middle SDI showing the most extreme position. High SDI regions are uniquely positioned near the axis intersection, reflecting their divergent response pattern.

Despite these substantial numerical patterns, it is important to note that individual ITS coefficient estimates did not achieve conventional statistical significance (all p-values >0.05; **Supplementary Table S3**). This lack of significance likely reflects multiple factors, including the short pandemic observation period, high within-region variability, and the inherent challenges of detecting acute impacts on chronic disease outcomes with long latency periods.

### 3.5 Intervention scenario modeling and preventable burden estimation

Based on our calculated population attributable fraction estimates (20.3%, 95% UI: 18.3-23.5%) using GBD 2021 comparative risk assessment methodology, intervention scenario modeling demonstrates substantial public health impact potential. Even modest 10% reductions in processed meat consumption could prevent approximately 2,330 deaths (95% UI: 1,600-3,100) and 124,000 DALYs (95% UI: 85,000-165,000) annually worldwide. A 25% reduction scenario would prevent an approximately d 5,800 deaths (95% UI: 4,000-7,750) and 310,000 DALYs (95% UI: 212,000-413,000), while a 50% reduction could approximately 11,600 deaths (95% UI: 8,000-15,500) and 620,000 DALYs (95% UI: 424,000-826,000) globally. The preventable burden varied substantially according to the SDI region. High SDI regions showed the largest absolute preventable burden under all scenarios, while low-middle SDI regions demonstrated the highest proportional reduction potential relative to baseline burden.

## 4 Discussion

This study reveals a significant increase in the global burden of type 2 diabetes mellitus attributable to high processed meat intake during the period from 1990 to 2021. The observed variations in the disease burden across different regions, genders, and age groups highlight the complexity of the relationship between processed meat consumption and type 2 diabetes mellitus and provide important evidence for global diabetes prevention strategies. The substantial global burden trends documented in our analysis can be explained through multiple interconnected pathogenic pathways linking processed meat consumption to T2DM development (**Supplementary Figure S6**). These mechanisms operate synergistically across different biological systems to impair glucose metabolism and insulin sensitivity. Processed meats contain high levels of preservatives such as sodium nitrite and sodium nitrate, which induce pancreatic β-cell apoptosis and impair insulin secretion, with effects that are more pronounced in male subjects due to sex-specific differences in nitric oxide metabolism. Advanced glycation end products (AGEs) formed during high-temperature processing accumulate in tissues to promote oxidative stress and systemic inflammation, particularly affecting postmenopausal women who show greater AGE-related metabolic dysregulation. Additionally, the high heme iron content disrupts gut microbiota composition, reducing beneficial short-chain fatty acid-producing bacteria, an effect that is most prominent in younger adults whose gut microbiome demonstrates higher plasticity. The convergence of these mechanistic pathways provides biological plausibility for the epidemiological associations observed in our global burden analysis and helps explain the demographic variations in disease burden patterns across age, sex, and regional contexts.

### 4.1 Trends in disease burden and underlying mechanisms

The results show that global deaths from type 2 diabetes mellitus attributed to high processed meat intake increased from 52.7 thousand in 1990 to 114.9 thousand in 2021, while DALYs increased from 2.02 million to 6.10 million, reflecting a persistent upward trend in this disease burden. This trend may be closely related to global dietary pattern transitions, increased consumption of processed meats, and population aging.

High intake of processed meat may contribute to the development of type 2 diabetes mellitus through multiple mechanisms. Processed meats often contain high levels of sodium, which can lead to hypertension and fluid retention, affecting insulin sensitivity (28). Additionally, the presence of preservatives and additives in processed meats, such as nitrates and nitrites, may generate harmful compounds during digestion that can disrupt metabolic processes and increase the risk of insulin resistance (29, 30). The high content of saturated fats and cholesterol in processed meats may also promote adipose tissue inflammation, further exacerbating insulin resistance (31). Experimental studies have shown that heme iron and advanced glycation end products (AGEs) in processed meats may interfere with insulin signaling pathways, increase oxidative stress, and damage pancreatic β-cell function (32). Furthermore, processed meat consumption may alter the gut microbiota, further aggravating insulin resistance (33). The combined effect of these mechanisms may explain the associations observed in epidemiological studies.

### 4.2 Regional disparities and their causes

This study reveals significant regional disparities in diabetes disease burden, with notable variations in ASMR and ASDR across different socio-demographic regions. In 2021, Southern Sub-Saharan Africa exhibited the highest ASMR at 3.6 per 100,000, while Oceania recorded the highest ASDR at 281.4 per 100,000. These regional differences underscore the complex interplay of multiple determinants, including dietary habits, healthcare accessibility, disease management, and socioeconomic development status. The divergent growth trajectories across SDI regions provide critical insights into the evolving diabetes landscape. High SDI regions, despite having relatively elevated ASMR and ASDR in both 1990 and 2021, demonstrated declining mortality trends (EAPC of ASMR: -1.56, 95% CI: -1.82 to -1.29) while showing modest increases in disability burden (EAPC of ASDR: 1.08, 95% CI: 0.96-1.20). Conversely, low-middle and middle SDI regions experienced the most rapid expansion, with EAPC of ASMR at 1.46 (95% CI: 1.39-1.52) and 1.13 (95% CI: 1.05-1.21), and EAPC of ASDR at 2.20 (95% CI: 2.16-2.24) and 1.95 (95% CI: 1.88-2.02), respectively.

The regional disparities in diabetes burden revealed in this study align closely with contemporary global nutrition transition theories. Existing research underscores the complex transformation occurring in developing countries, where dietary patterns are shifting from traditional to Western models, reflecting a profound intersection of socioeconomic development and nutritional change (34). Recent studies have highlighted how rapid economic development accelerates the adoption of Western dietary patterns, particularly increased processed food consumption in urbanizing regions (35). The divergent growth rates across different Socio-demographic Index (SDI) regions illuminate the non-linear relationship between economic development and nutritional transition. The significantly higher growth rates in low-middle and middle SDI regions, compared to high SDI regions, substantiate theoretical insights into the potential misalignment between rapid economic progression and public health system responsiveness (36). This disparity is particularly evident in the context of accelerated urbanization, where rapidly evolving dietary habits outpace corresponding public health interventions (37).

This study further corroborates the substantial impact of nutritional transition on metabolic disease risks. As economic development progresses, increased processed food consumption and sedentary lifestyles create a fertile ground for chronic metabolic diseases like diabetes (38). This phenomenon is particularly pronounced in developing countries, reflecting the profound health implications of socioeconomic transformations.

### 4.3 Age patterns and gender differences

This study found that the burden of type 2 diabetes mellitus deaths peaked in the 70-74 age group, while the DALYs burden peaked in the 65-69 age group. This age-related increase in disease burden can be attributed to several factors. With advancing age, there is a decline in physical activity levels, which, combined with higher consumption of processed meats, can lead to weight gain and an increased risk of metabolic disorders. Recent research has demonstrated that age-related changes in intestinal microbiota composition may amplify the negative metabolic effects of processed meat consumption, creating greater vulnerability in elderly populations (39). The study also shows that females had a higher overall burden than males, although the gender gap has been narrowing. Gender differences in disease burden may be influenced by various factors, including differences in dietary patterns, lifestyle behaviors, and hormonal factors. Women may be more affected by the hormonal changes associated with menopause, which can increase insulin resistance (12). Moreover, societal norms and roles may lead to differences in processed meat consumption and physical activity levels between men and women.

Previous research has emphasized the gender differences in type 2 diabetes mellitus risk, pathophysiology, and complications, which is consistent with our findings. However, the trend of a narrowing gender gap that we observed may reflect the gradual convergence of gender roles and dietary behaviors, a point that has been less reported in previous studies. Recent epidemiological research has demonstrated progressive equalization of processed meat consumption patterns between genders, particularly in high and middle-income regions, possibly explaining this convergence in disease burden (40).

### 4.4 Relationship between SDI and disease burden

The relationship between SDI and the burden of type 2 diabetes mellitus due to high processed meat intake reflects complex socioeconomic determinants. In low SDI regions, limited access to healthy food options, affordability of processed meats, and insufficient physical activity contribute to higher disease burden. Economic transitions in low- and middle-income countries have increased ultra-processed food availability, including processed meats, accelerating type 2 diabetes mellitus prevalence (41). Food environment inequalities function as structural determinants of nutritional disparities, with processed foods often more accessible and affordable than fresh alternatives. High SDI regions demonstrate relatively lower burden growth, potentially due to better healthcare access and dietary awareness. However, these regions face substantial challenges from the ultra-processed food industry expansion and targeted marketing. Ultra-processed food consumption significantly increases caloric intake and metabolic disturbances even among informed populations, suggesting that processed meat reduction requires structural interventions beyond individual awareness (42). The observed stabilization of diabetes prevalence growth in high-income countries contrasts with rapid increases in middle and low-income countries. Middle-income countries currently exhibit the fastest increases in processed meat consumption globally, correlating with rising diabetes incidence rates (43). Inadequate regulatory policies addressing food composition, availability, and marketing substantially contribute to the growing burden of diet-related non-communicable diseases across development contexts (40).

Our advanced statistical modeling revealed a critical finding that challenges previous assumptions about linear relationships between socioeconomic development and disease burden trends. The identification of a quadratic relationship between SDI and EAPC values, with peak burden growth occurring at SDI values of 0.468 (mortality) and 0.549 (disability), provides novel insights into the nutrition transition epidemiology. This finding aligns with recent theoretical proposals that middle-income countries experience peak vulnerability during rapid dietary pattern shifts (36). The critical SDI range of 0.47-0.55 identified in our analysis corresponds to countries experiencing rapid economic development with insufficient regulatory infrastructure for food policy implementation. Recent evidence demonstrates that countries in this development phase show the steepest increases in ultra-processed food consumption, supporting our empirical findings (44). The validation through GBD quintile analysis, showing low-middle SDI countries with the highest mean EAPC (1.47 ± 1.24% per year), provides robust evidence for targeted intervention prioritization in middle-income contexts.

This study on the attributable burden of processed meat to type 2 diabetes mellitus demonstrates significant regional variation resulting from differences in consumption patterns, genetic factors, and comorbidity profile (45). The weak correlation between SDI and EAPC of ASDR, contrasted with a stronger correlation for ASMR, suggests differential impacts of development on mortality versus overall disease burden. These findings indicate that intervention strategies must account for both regional context and development trajectory to effectively address this modifiable dietary risk factor.

### 4.5 Impact of COVID-19 on high intake of processed meat and type 2 diabetes mellitus

While ITS analysis did not achieve statistical significance, the observed patterns suggest the COVID-19 pandemic may have revealed vulnerabilities in the global health system’s capacity to maintain chronic disease management during acute crises. While our interrupted time series analysis did not achieve statistical significance (p>0.05), the strong positive correlation (r = 0.920) between mortality and disability responses warrants epidemiological consideration, particularly given the inherent challenges of detecting pandemic impacts on diseases with long latency periods. The non-significance of individual effects likely reflects methodological rather than epidemiological realities.

An unexpected finding was the divergent response patterns across SDI strata. High SDI regions’ ability to maintain mortality outcomes while experiencing disability increases suggests that technological interventions—telemedicine, continuous monitoring, AI-assisted management—may effectively prevent acute complications but fail to address the behavioral and metabolic determinants underlying disease progression (46, 47). This raises important questions about the sustainability and completeness of technology-dependent care models. Conversely, the pronounced effects observed in middle-income countries highlight a different vulnerability profile. These nations, undergoing rapid nutrition transition, may lack the regulatory frameworks and health system redundancy to buffer against acute disruptions (48). The pandemic essentially accelerated pre-existing trajectories rather than creating novel patterns, suggesting that crisis preparedness must account for baseline vulnerabilities rather than assuming uniform response capabilities (49). The persistence of regional burden hierarchies despite pandemic perturbations challenges assumptions about health system adaptability. If acute crises cannot disrupt fundamental health inequalities, this implies that addressing diet-related disease burden requires structural interventions targeting underlying socioeconomic determinants rather than health system strengthening alone (50). The pandemic served as a natural experiment, revealing which aspects of health systems are resilient (acute care in high-income settings) versus vulnerable (prevention services universally). Perhaps most concerning is evidence suggesting pandemic dietary changes may persist beyond the acute crisis period (51, 52). Data from multiple cohorts show that HbA1c levels remained significantly elevated during the COVID-19 period, with concurrent increases in total cholesterol and triglyceride levels (53). If confirmed through longer-term surveillance, this would represent a step-change in population-level diabetes risk that could manifest as increased burden throughout the 2020s. This possibility underscores the importance of proactive nutritional interventions during crisis periods rather than assuming automatic behavioral reversion post-crisis.

Our findings highlight a critical gap in pandemic preparedness frameworks, which traditionally prioritize infectious disease response over chronic disease management continuity. The synchronized disruption across mortality and disability outcomes suggests that future preparedness must adopt a “dual-track” approach, maintaining both acute surge capacity and routine chronic care capabilities (54). This requires fundamental reimagining of health system design, moving from segregated vertical programs toward integrated platforms capable of maintaining essential services across the disease spectrum during crises.

### 4.6 Policy implementation considerations and real-world barriers

The substantial burden and regional disparities demonstrated above highlight the urgent need for effective intervention strategies. Based on our calculated population attributable fraction estimates (20.3%, 95% UI: 18.3-23.5%) using GBD 2021 comparative risk assessment methodology, our intervention scenario modeling demonstrates substantial public health impact potential, with even modest 10% reductions in processed meat consumption preventing approximately 2,330 deaths (95% UI: 1,600-3,100) and 124,000 DALYs (95% UI: 85,000-165,000) annually worldwide. However, successful implementation faces significant real-world challenges that extend far beyond individual dietary education. Cultural preferences and dietary traditions represent substantial obstacles, particularly in regions where processed meats are deeply embedded in traditional cuisines and social practices (55). The processed food industry’s sophisticated marketing strategies, often targeting vulnerable populations through carefully designed campaigns, may systematically counteract public health messaging efforts (56).

Effective population-level reduction requires comprehensive policy frameworks addressing structural determinants of food choice. Evidence from successful tobacco control policies suggests that coordinated approaches combining regulatory, economic, and social strategies yield the greatest impact (57). Regulatory interventions should include mandatory health warning labels on processed meat products, restrictions on marketing to children, and taxation policies that alter relative costs between processed and fresh alternatives, following successful models implemented in Chile’s comprehensive food labeling system and processed meat taxes in Denmark (26, 27). Environmental and economic incentives, including subsidies for fresh food retailers and urban planning improvements enhancing access to whole foods in underserved areas, complement regulatory approaches and have demonstrated effectiveness in improving dietary quality among low-income populations (58). Our identification of critical SDI ranges (0.47-0.55) where burden growth peaks suggests that middle-income countries require particularly targeted intervention approaches that can adapt to rapid nutrition transition dynamics while building regulatory infrastructure. These nations experience vulnerable windows during which processed food consumption patterns become entrenched, yet early intervention during nutrition transition periods can significantly alter long-term dietary trajectories (34). High-SDI regions may benefit from market-based approaches leveraging existing consumer awareness, while low-SDI regions require structural interventions focusing on food access and supply chain improvements (59). Healthcare system integration through clinical guidelines, incorporating processed meat reduction counseling and population-level screening programs in high-risk regions, represents a critical but underutilized intervention opportunity.

Policymakers must carefully balance public health benefits with economic impacts on food industry employment and address legitimate social equity concerns about food choice restrictions. Community engagement strategies are essential to ensure cultural sensitivity and population acceptance, particularly given the significant variation in food preferences across ethnic and socioeconomic groups (60). The COVID-19 pandemic’s impact on dietary patterns, as demonstrated in our interrupted time series analysis and confirmed by longitudinal studies showing persistent increases in processed food consumption and decreased physical activity, underscores the importance of building resilient food policy systems that maintain effectiveness during crisis periods (61, 62). Immediate policy priorities should focus on implementing comprehensive interventions in middle-income countries experiencing rapid nutrition transitions, developing culturally-adapted strategies for high-burden regions, and establishing monitoring systems to track policy effectiveness and adapt interventions based on real-world outcomes.

### 4.7 Study limitations

Several methodological limitations merit acknowledgment. The GBD 2021 framework’s reliance on diverse dietary assessment methodologies introduces systematic biases, including well-documented underreporting of processed food consumption by 12-23% in validation studies. The harmonization of 305 heterogeneous data sources across countries and periods, while improving global coverage, may mask important cross-country variations in dietary measurement approaches. Unmeasured confounding represents a significant limitation, as we could not control for individual-level factors, including physical activity, overall dietary quality, and genetic predisposition that may substantially modify the processed meat-T2DM relationship. The intervention scenario modeling was constrained by the absence of population-specific dose-response data, limiting precise quantitative projections under specific intervention scenarios. Finally, residual uncertainty in low- and middle-SDI regions due to sparse surveillance infrastructure may limit the precision of trend estimates in these populations, despite their critical importance for global health policy. These limitations highlight the trade-offs between global comprehensiveness and analytical granularity inherent in secondary analysis of international surveillance data.

## 5 Conclusions

Our identification of the critical SDI range (0.47-0.55), where processed meat-attributable diabetes burden peaks, provides actionable evidence for targeted interventions. Policymakers in middle-income countries should implement comprehensive strategies, including front-of-package warning labels, taxation policies that increase processed meat costs while subsidizing alternatives, and integration of reduction counseling into national diabetes programs. Even modest 10% consumption reductions could prevent 2,330 deaths and 124,000 DALYs annually worldwide. Healthcare providers should prioritize processed meat reduction counseling for high-risk patients during routine screening, particularly in middle-income settings. The pandemic-induced mortality-disability divergence highlights the need for enhanced chronic disease management protocols that maintain care continuity during healthcare disruptions. Critical research priorities include prospective cohort studies in underrepresented middle-income populations to validate individual-level associations, randomized controlled trials testing culturally-adapted interventions with clinical outcomes, and continued surveillance through 2030 to assess long-term pandemic dietary impacts. The interrupted time series methodology demonstrated here provides a framework for assessing how global emergencies influence chronic disease patterns, enabling real-time policy adjustments. The convergence of evidence across 204 countries provides unprecedented justification for immediate action targeting processed meat consumption, with clear prioritization for middle-income countries experiencing nutrition transition vulnerability.

## Data Availability

The original contributions presented in the study are included in the article/**Supplementary Material**. Further inquiries can be directed to the corresponding authors.
