# Supplementary material for: A systematic analysis of the global disease burden of type 2 diabetes mellitus attributable to high intake of processed meat in 204 countries (1990-2021)
Source: Front Endocrinol (Lausanne). 2025 Sep 30;16:1635831. doi: 10.3389/fendo.2025.1635831 (PMC12518099; doi:10.3389/fendo.2025.1635831)
Supplement: Supplementary file 1 [file DataSheet1.docx]

***Supplementary Tables***

**Supplementary TABLE 1.**Deaths and ASMR of T2D attributable to high Intake of processed meat across 204 countries and territories

| Country/Territory | Death cases, n × 10³ (95% UI) (1990) | ASMR per 10⁵, n (95% UI) (2021) | Death cases, n × 10³ (95% UI) (2021) | ASMR per 10⁵, n (95% UI) (2021) | EAPC ASMR, n (95% CI) (1990-2021) |
| --- | --- | --- | --- | --- | --- |
| Bosnia and Herzegovina | 0.08 (0.02-0.13) | 1.78 (0.41-2.96) | 0.31 (0.07-0.54) | 9.46 (2.15-16.49) | 4.72 (4.06-5.39) |
| Malta | 0.02 (0.01-0.03) | 5.61 (1.35-9.00) | 0.03 (0.01-0.05) | 6.93 (1.68-11.04) | -0.67 (-1.43-0.09) |
| North Macedonia | 0.05 (0.01-0.09) | 2.72 (0.67-4.65) | 0.15 (0.03-0.26) | 6.89 (1.56-11.74) | 2.52 (2.17-2.88) |
| Latvia | 0.04 (0.01-0.07) | 1.68 (0.41-2.66) | 0.12 (0.03-0.20) | 6.66 (1.56-10.83) | 3.38 (2.44-4.33) |
| Trinidad and Tobago | 0.05 (0.01-0.08) | 3.99 (0.93-6.97) | 0.09 (0.02-0.16) | 6.59 (1.49-11.76) | 0.29 (-0.04-0.62) |
| Czechia | 0.30 (0.07-0.49) | 2.89 (0.70-4.75) | 0.69 (0.17-1.18) | 6.46 (1.56-11.08) | 3.91 (2.90-4.93) |
| Fiji | 0.02 (0.00-0.03) | 2.31 (0.53-4.03) | 0.06 (0.01-0.10) | 6.23 (1.31-11.19) | 2.26 (1.45-3.08) |
| Cyprus | 0.07 (0.02-0.12) | 9.57 (2.40-15.82) | 0.08 (0.02-0.14) | 6.22 (1.47-10.41) | -2.74 (-3.01--2.48) |
| Bulgaria | 0.32 (0.08-0.53) | 3.71 (0.87-6.06) | 0.42 (0.09-0.68) | 6.13 (1.39-10.07) | 0.66 (-0.17-1.50) |
| Italy | 3.35 (0.81-5.46) | 5.89 (1.42-9.62) | 3.60 (0.84-5.87) | 6.01 (1.40-9.81) | -0.74 (-1.86-0.40) |
| Cook Islands | 0.00 (0.00-0.00) | 2.66 (0.60-4.61) | 0.00 (0.00-0.00) | 5.61 (1.22-9.84) | 0.94 (0.56-1.32) |
| Serbia | 0.27 (0.06-0.45) | 2.85 (0.66-4.69) | 0.50 (0.12-0.85) | 5.59 (1.36-9.56) | 1.03 (0.41-1.64) |
| Russian Federation | 1.59 (0.39-2.50) | 1.05 (0.26-1.66) | 7.80 (1.84-12.47) | 5.39 (1.27-8.61) | 3.64 (2.42-4.88) |
| Germany | 4.21 (1.01-6.90) | 5.27 (1.26-8.63) | 4.41 (1.02-7.23) | 5.16 (1.19-8.47) | -1.08 (-2.12--0.04) |
| Portugal | 0.37 (0.08-0.63) | 3.69 (0.83-6.17) | 0.54 (0.13-0.92) | 5.05 (1.21-8.69) | -0.61 (-1.57-0.37) |
| Montenegro | 0.01 (0.00-0.02) | 2.12 (0.47-3.43) | 0.03 (0.01-0.05) | 5.01 (1.23-8.53) | 2.22 (1.80-2.65) |
| Uruguay | 0.11 (0.03-0.18) | 3.46 (0.80-5.69) | 0.17 (0.04-0.28) | 5.01 (1.22-8.11) | 0.59 (0.02-1.16) |
| Estonia | 0.02 (0.00-0.03) | 1.14 (0.27-1.81) | 0.07 (0.02-0.11) | 5.00 (1.23-8.23) | 2.89 (1.86-3.94) |
| Hungary | 0.29 (0.07-0.48) | 2.78 (0.66-4.61) | 0.48 (0.12-0.76) | 4.97 (1.20-7.92) | 1.70 (0.88-2.54) |
| Niue | 0.00 (0.00-0.00) | 2.24 (0.51-3.99) | 0.00 (0.00-0.00) | 4.81 (1.14-8.89) | 2.09 (1.78-2.39) |

**Supplementary TABLE 1. (Continued)**

| Country/Territory | Death cases, n × 10³ (95% UI) (1990) | ASMR per 10⁵, n (95% UI) (2021) | Death cases, n × 10³ (95% UI) (2021) | ASMR per 10⁵, n (95% UI) (2021) | EAPC ASMR, n (95% CI) (1990-2021) |
| --- | --- | --- | --- | --- | --- |
| Denmark | 0.16 (0.04-0.26) | 3.09 (0.73-5.03) | 0.27 (0.06-0.45) | 4.64 (1.09-7.62) | 0.80 (-0.14-1.75) |
| Lithuania | 0.03 (0.01-0.05) | 0.94 (0.24-1.49) | 0.12 (0.03-0.21) | 4.57 (1.12-7.52) | 3.32 (2.29-4.35) |
| Barbados | 0.01 (0.00-0.02) | 4.08 (0.88-7.09) | 0.01 (0.00-0.02) | 4.57 (1.01-8.10) | -0.50 (-0.97--0.02) |
| United States of America | 9.50 (2.26-15.23) | 3.74 (0.89-6.00) | 15.13 (3.73-24.27) | 4.55 (1.12-7.30) | -0.61 (-1.24-0.02) |
| Puerto Rico | 0.08 (0.02-0.13) | 2.20 (0.48-3.73) | 0.14 (0.03-0.25) | 4.31 (0.98-7.62) | 0.42 (-0.22-1.06) |
| Austria | 0.32 (0.08-0.51) | 4.12 (1.01-6.60) | 0.38 (0.09-0.61) | 4.18 (0.99-6.84) | 0.28 (-0.72-1.29) |
| Georgia | 0.09 (0.02-0.15) | 1.71 (0.42-2.80) | 0.15 (0.03-0.25) | 4.15 (0.95-7.01) | 3.52 (2.76-4.28) |
| Slovenia | 0.05 (0.01-0.09) | 2.73 (0.66-4.44) | 0.08 (0.02-0.14) | 4.10 (0.96-6.62) | -1.57 (-2.46--0.68) |
| Dominica | 0.00 (0.00-0.00) | 2.49 (0.56-4.44) | 0.00 (0.00-0.00) | 4.06 (0.97-7.43) | 0.64 (0.46-0.82) |
| Spain | 2.03 (0.48-3.22) | 5.23 (1.25-8.29) | 1.81 (0.45-2.98) | 3.97 (0.99-6.55) | -2.14 (-3.11--1.16) |
| Sweden | 0.30 (0.07-0.48) | 3.48 (0.83-5.64) | 0.41 (0.10-0.66) | 3.93 (0.94-6.38) | 0.08 (-1.04-1.21) |
| Mexico | 1.65 (0.39-2.79) | 1.93 (0.46-3.27) | 5.05 (1.24-9.05) | 3.91 (0.96-7.00) | 1.03 (0.36-1.71) |
| France | 1.58 (0.37-2.60) | 2.74 (0.63-4.49) | 2.58 (0.63-4.24) | 3.88 (0.94-6.39) | 0.25 (-0.78-1.28) |
| Brunei Darussalam | 0.01 (0.00-0.01) | 3.12 (0.77-5.52) | 0.02 (0.00-0.03) | 3.80 (0.94-6.35) | -0.07 (-1.23-1.11) |
| Israel | 0.13 (0.03-0.22) | 2.64 (0.59-4.41) | 0.36 (0.09-0.62) | 3.80 (0.89-6.42) | -0.05 (-0.67-0.58) |
| Saint Vincent and the Grenadines | 0.00 (0.00-0.00) | 2.30 (0.52-4.04) | 0.00 (0.00-0.01) | 3.78 (0.86-6.72) | 0.32 (-0.09-0.73) |
| Palau | 0.00 (0.00-0.00) | 1.45 (0.35-2.54) | 0.00 (0.00-0.00) | 3.78 (0.83-6.84) | 2.29 (1.82-2.76) |
| South Africa | 0.41 (0.09-0.69) | 1.10 (0.25-1.87) | 1.98 (0.46-3.40) | 3.48 (0.81-5.98) | 3.77 (3.00-4.55) |
| Jamaica | 0.05 (0.01-0.09) | 2.25 (0.53-3.97) | 0.10 (0.02-0.17) | 3.40 (0.78-5.97) | 0.36 (0.06-0.65) |
| Mauritius | 0.01 (0.00-0.01) | 0.68 (0.14-1.20) | 0.04 (0.01-0.07) | 3.39 (0.76-5.88) | 5.70 (4.91-6.50) |
| American Samoa | 0.00 (0.00-0.00) | 1.23 (0.27-2.10) | 0.00 (0.00-0.00) | 3.38 (0.78-6.08) | 2.05 (1.23-2.88) |
| Netherlands | 0.66 (0.15-1.08) | 4.42 (1.01-7.21) | 0.58 (0.13-0.93) | 3.35 (0.77-5.39) | -1.86 (-2.65--1.07) |

**Supplementary TABLE 1. (Continued)**

| Country/Territory | Death cases, n × 10³ (95% UI) (1990) | ASMR per 10⁵, n (95% UI) (2021) | Death cases, n × 10³ (95% UI) (2021) | ASMR per 10⁵, n (95% UI) (2021) | EAPC ASMR, n (95% CI) (1990-2021) |
| --- | --- | --- | --- | --- | --- |
| Poland | 0.60 (0.14-1.03) | 1.57 (0.37-2.71) | 1.27 (0.30-2.09) | 3.33 (0.79-5.46) | 2.23 (1.58-2.89) |
| Guyana | 0.01 (0.00-0.02) | 1.55 (0.36-2.69) | 0.03 (0.01-0.05) | 3.31 (0.69-6.14) | 1.44 (0.62-2.26) |
| Andorra | 0.00 (0.00-0.00) | 2.29 (0.57-3.85) | 0.00 (0.00-0.00) | 3.30 (0.82-5.53) | 0.23 (-0.36-0.83) |
| Grenada | 0.00 (0.00-0.00) | 2.61 (0.57-4.67) | 0.00 (0.00-0.01) | 3.21 (0.69-5.69) | 0.49 (0.13-0.86) |
| Taiwan (Province of China) | 0.27 (0.06-0.47) | 1.34 (0.29-2.30) | 0.75 (0.16-1.32) | 3.18 (0.69-5.57) | 0.50 (-0.04-1.03) |
| Chile | 0.28 (0.07-0.46) | 2.14 (0.52-3.48) | 0.59 (0.15-0.94) | 3.14 (0.78-5.01) | 0.40 (-0.00-0.81) |
| Australia | 0.39 (0.09-0.63) | 2.31 (0.56-3.76) | 0.81 (0.19-1.34) | 3.13 (0.74-5.19) | -0.15 (-0.80-0.50) |
| Saint Lucia | 0.00 (0.00-0.01) | 2.23 (0.50-3.84) | 0.01 (0.00-0.01) | 3.02 (0.70-5.48) | -1.29 (-1.78--0.80) |
| Bhutan | 0.01 (0.00-0.01) | 0.86 (0.20-1.53) | 0.02 (0.01-0.04) | 2.99 (0.67-5.33) | 2.75 (1.76-3.75) |
| Uzbekistan | 0.16 (0.04-0.27) | 0.77 (0.18-1.27) | 0.99 (0.22-1.72) | 2.90 (0.65-5.04) | 3.43 (2.65-4.22) |
| Argentina | 0.84 (0.20-1.40) | 2.52 (0.59-4.22) | 1.31 (0.32-2.19) | 2.89 (0.71-4.82) | -0.12 (-0.33-0.09) |
| Venezuela (Bolivarian Republic of) | 0.17 (0.04-0.29) | 0.89 (0.20-1.52) | 0.77 (0.16-1.37) | 2.89 (0.61-5.13) | 2.56 (1.82-3.30) |
| Kiribati | 0.00 (0.00-0.00) | 1.66 (0.39-2.94) | 0.00 (0.00-0.01) | 2.88 (0.65-5.47) | 1.53 (0.54-2.52) |
| United States Virgin Islands | 0.00 (0.00-0.00) | 1.37 (0.30-2.42) | 0.00 (0.00-0.00) | 2.88 (0.64-5.31) | 0.69 (0.19-1.19) |
| Canada | 0.68 (0.17-1.12) | 2.51 (0.62-4.13) | 1.07 (0.24-1.74) | 2.85 (0.65-4.63) | -1.18 (-1.98--0.38) |
| Turkmenistan | 0.03 (0.01-0.05) | 0.78 (0.19-1.30) | 0.15 (0.04-0.26) | 2.83 (0.70-4.99) | 2.92 (2.12-3.73) |
| Marshall Islands | 0.00 (0.00-0.00) | 0.91 (0.20-1.69) | 0.00 (0.00-0.00) | 2.81 (0.64-5.64) | 2.82 (1.54-4.12) |
| Greece | 0.18 (0.04-0.31) | 1.78 (0.41-2.97) | 0.28 (0.07-0.46) | 2.78 (0.66-4.56) | 0.53 (-0.44-1.51) |
| Lesotho | 0.02 (0.00-0.03) | 1.05 (0.24-1.82) | 0.05 (0.01-0.10) | 2.78 (0.67-5.11) | 4.10 (3.13-5.09) |
| Republic of Moldova | 0.06 (0.01-0.09) | 1.25 (0.31-2.04) | 0.10 (0.02-0.16) | 2.76 (0.69-4.53) | 0.63 (-0.13-1.39) |
| Gabon | 0.02 (0.01-0.04) | 2.21 (0.51-3.97) | 0.05 (0.01-0.09) | 2.73 (0.63-4.90) | 0.64 (-0.31-1.59) |
| Slovakia | 0.11 (0.03-0.18) | 2.02 (0.49-3.37) | 0.15 (0.04-0.25) | 2.72 (0.68-4.55) | 0.42 (-0.07-0.91) |

**Supplementary TABLE 1. (Continued)**

| Country/Territory | Death cases, n × 10³ (95% UI) (1990) | ASMR per 10⁵, n (95% UI) (2021) | Death cases, n × 10³ (95% UI) (2021) | ASMR per 10⁵, n (95% UI) (2021) | EAPC ASMR, n (95% CI) (1990-2021) |
| --- | --- | --- | --- | --- | --- |
| Characteristic | Death cases, n × 10³ (95% UI) (1990) | ASMR per 10⁵, n (95% UI) (2021) | Death cases, n × 10³ (95% UI) (2021) | ASMR per 10⁵, n (95% UI) (2021) | EAPC ASMR, n (95% CI) (1990-2021) |
| Tokelau | 0.00 (0.00-0.00) | 1.58 (0.36-2.91) | 0.00 (0.00-0.00) | 2.69 (0.58-4.94) | 1.37 (1.17-1.58) |
| Bangladesh | 1.11 (0.27-1.94) | 1.02 (0.24-1.78) | 4.42 (1.07-7.58) | 2.68 (0.65-4.60) | 2.11 (0.86-3.37) |
| Nepal | 0.19 (0.04-0.33) | 0.95 (0.22-1.68) | 0.83 (0.19-1.45) | 2.66 (0.60-4.66) | 2.89 (1.89-3.89) |
| Cabo Verde | 0.00 (0.00-0.00) | 0.65 (0.15-1.13) | 0.01 (0.00-0.03) | 2.66 (0.58-4.65) | 3.90 (3.24-4.55) |
| Romania | 0.28 (0.07-0.45) | 1.18 (0.30-1.94) | 0.49 (0.11-0.81) | 2.59 (0.59-4.29) | 2.03 (1.31-2.75) |
| Azerbaijan | 0.08 (0.02-0.13) | 1.05 (0.24-1.78) | 0.27 (0.06-0.48) | 2.56 (0.55-4.58) | 2.14 (1.67-2.61) |
| Switzerland | 0.29 (0.07-0.47) | 4.24 (0.97-6.89) | 0.23 (0.05-0.37) | 2.53 (0.58-4.16) | -2.69 (-3.66--1.70) |
| Belgium | 0.35 (0.09-0.58) | 3.54 (0.86-5.77) | 0.29 (0.07-0.46) | 2.51 (0.61-4.04) | -1.85 (-2.79--0.90) |
| Ghana | 0.14 (0.03-0.24) | 0.94 (0.23-1.60) | 0.85 (0.19-1.47) | 2.48 (0.56-4.28) | 3.48 (2.18-4.79) |
| Norway | 0.11 (0.03-0.17) | 2.53 (0.61-4.03) | 0.13 (0.03-0.21) | 2.44 (0.59-3.92) | -0.66 (-1.63-0.31) |
| Pakistan | 1.59 (0.40-2.80) | 1.43 (0.36-2.52) | 5.67 (1.37-9.89) | 2.41 (0.58-4.20) | 1.36 (0.21-2.53) |
| Eswatini | 0.01 (0.00-0.02) | 1.10 (0.26-1.93) | 0.03 (0.01-0.05) | 2.38 (0.59-4.47) | 2.49 (1.01-3.98) |
| Nauru | 0.00 (0.00-0.00) | 1.56 (0.33-2.77) | 0.00 (0.00-0.00) | 2.34 (0.54-4.32) | 0.97 (-0.06-2.01) |
| Tonga | 0.00 (0.00-0.00) | 1.24 (0.29-2.27) | 0.00 (0.00-0.00) | 2.33 (0.50-4.17) | 1.49 (0.85-2.14) |
| New Zealand | 0.07 (0.02-0.12) | 2.12 (0.49-3.52) | 0.12 (0.03-0.20) | 2.33 (0.56-3.79) | -1.14 (-1.71--0.56) |
| Monaco | 0.00 (0.00-0.00) | 1.93 (0.47-3.22) | 0.00 (0.00-0.00) | 2.30 (0.53-3.83) | 0.34 (-1.10-1.80) |
| Luxembourg | 0.01 (0.00-0.02) | 3.26 (0.77-5.19) | 0.01 (0.00-0.02) | 2.28 (0.54-3.65) | -1.47 (-2.11--0.82) |
| Antigua and Barbuda | 0.00 (0.00-0.00) | 1.96 (0.51-3.37) | 0.00 (0.00-0.00) | 2.26 (0.53-3.95) | -0.22 (-0.50-0.06) |
| Republic of Korea | 0.58 (0.14-0.98) | 1.32 (0.31-2.21) | 1.15 (0.28-1.93) | 2.24 (0.53-3.75) | -0.52 (-1.26-0.23) |
| Tuvalu | 0.00 (0.00-0.00) | 1.43 (0.32-2.60) | 0.00 (0.00-0.00) | 2.24 (0.50-4.10) | 1.11 (0.71-1.52) |
| San Marino | 0.00 (0.00-0.00) | 2.47 (0.57-3.99) | 0.00 (0.00-0.00) | 2.23 (0.59-3.82) | -0.58 (-1.75-0.60) |

**Supplementary TABLE 1. (Continued)**

| Country/Territory | Death cases, n × 10³ (95% UI) (1990) | ASMR per 10⁵, n (95% UI) (2021) | Death cases, n × 10³ (95% UI) (2021) | ASMR per 10⁵, n (95% UI) (2021) | EAPC ASMR, n (95% CI) (1990-2021) |
| --- | --- | --- | --- | --- | --- |
| Armenia | 0.05 (0.01-0.08) | 1.34 (0.32-2.28) | 0.07 (0.02-0.11) | 2.20 (0.54-3.64) | 0.57 (-0.25-1.39) |
| Guatemala | 0.03 (0.01-0.04) | 0.30 (0.07-0.52) | 0.34 (0.07-0.60) | 2.18 (0.46-3.80) | 4.42 (3.29-5.56) |
| Paraguay | 0.02 (0.01-0.04) | 0.60 (0.13-1.05) | 0.16 (0.03-0.28) | 2.18 (0.48-3.97) | 3.88 (3.17-4.60) |
| Senegal | 0.09 (0.02-0.16) | 1.21 (0.29-2.04) | 0.34 (0.09-0.60) | 2.17 (0.54-3.76) | 1.85 (0.60-3.12) |
| Northern Mariana Islands | 0.00 (0.00-0.00) | 0.81 (0.19-1.42) | 0.00 (0.00-0.00) | 2.16 (0.48-3.85) | 1.87 (0.81-2.95) |
| Micronesia (Federated States of) | 0.00 (0.00-0.00) | 0.95 (0.20-1.71) | 0.00 (0.00-0.00) | 2.14 (0.45-4.04) | 2.01 (1.05-2.99) |
| Suriname | 0.00 (0.00-0.01) | 1.10 (0.25-1.85) | 0.01 (0.00-0.02) | 2.14 (0.53-3.88) | 1.63 (1.20-2.06) |
| Greenland | 0.00 (0.00-0.00) | 1.59 (0.39-2.55) | 0.00 (0.00-0.00) | 2.12 (0.52-3.39) | -0.36 (-0.87-0.15) |
| Saint Kitts and Nevis | 0.00 (0.00-0.00) | 2.60 (0.55-4.69) | 0.00 (0.00-0.00) | 2.11 (0.47-3.74) | -0.62 (-0.95--0.29) |
| El Salvador | 0.03 (0.01-0.05) | 0.54 (0.13-0.92) | 0.14 (0.03-0.24) | 2.10 (0.48-3.78) | 3.46 (2.93-3.99) |
| Brazil | 1.28 (0.29-2.21) | 0.86 (0.20-1.49) | 4.57 (1.03-7.87) | 2.07 (0.47-3.57) | 2.11 (1.64-2.59) |
| Samoa | 0.00 (0.00-0.00) | 1.00 (0.22-1.76) | 0.00 (0.00-0.01) | 1.93 (0.45-3.47) | 1.74 (0.94-2.56) |
| Finland | 0.09 (0.02-0.14) | 1.74 (0.41-2.84) | 0.11 (0.03-0.17) | 1.93 (0.46-3.14) | -0.89 (-1.83-0.06) |
| United Kingdom | 1.70 (0.41-2.75) | 2.96 (0.72-4.80) | 1.29 (0.31-2.08) | 1.90 (0.46-3.07) | -1.90 (-2.79--1.01) |
| Cameroon | 0.15 (0.04-0.25) | 1.43 (0.34-2.41) | 0.60 (0.15-1.08) | 1.89 (0.47-3.40) | 1.00 (-0.43-2.45) |
| Mauritania | 0.03 (0.01-0.05) | 1.43 (0.34-2.41) | 0.08 (0.02-0.15) | 1.88 (0.41-3.52) | 0.72 (-0.45-1.90) |
| Congo | 0.04 (0.01-0.07) | 1.57 (0.35-2.79) | 0.09 (0.03-0.17) | 1.73 (0.47-3.07) | -0.08 (-1.31-1.17) |
| Gambia | 0.01 (0.00-0.01) | 0.86 (0.21-1.48) | 0.04 (0.01-0.07) | 1.69 (0.38-2.96) | 1.93 (0.48-3.40) |
| Bahrain | 0.00 (0.00-0.01) | 0.90 (0.20-1.58) | 0.03 (0.01-0.05) | 1.68 (0.36-3.00) | 0.78 (-1.00-2.60) |
| Belize | 0.00 (0.00-0.00) | 0.91 (0.21-1.63) | 0.01 (0.00-0.01) | 1.65 (0.37-2.90) | 1.32 (0.37-2.28) |
| Croatia | 0.03 (0.01-0.05) | 0.62 (0.15-1.09) | 0.07 (0.01-0.12) | 1.64 (0.35-2.85) | 1.48 (0.60-2.37) |
| Comoros | 0.01 (0.00-0.01) | 1.10 (0.24-1.92) | 0.01 (0.00-0.02) | 1.64 (0.35-3.00) | 0.44 (-0.62-1.51) |

**Supplementary TABLE 1. (Continued)**

| Country/Territory | Death cases, n × 10³ (95% UI) (1990) | ASMR per 10⁵, n (95% UI) (2021) | Death cases, n × 10³ (95% UI) (2021) | ASMR per 10⁵, n (95% UI) (2021) | EAPC ASMR, n (95% CI) (1990-2021) |
| --- | --- | --- | --- | --- | --- |
| Côte d'Ivoire | 0.12 (0.03-0.20) | 0.96 (0.22-1.64) | 0.46 (0.11-0.84) | 1.64 (0.40-3.01) | 1.33 (-0.31-2.99) |
| Albania | 0.02 (0.00-0.03) | 0.55 (0.14-0.91) | 0.04 (0.01-0.07) | 1.63 (0.42-2.67) | 2.04 (1.51-2.57) |
| Guinea | 0.09 (0.02-0.15) | 1.46 (0.34-2.53) | 0.22 (0.06-0.40) | 1.63 (0.41-2.96) | 1.03 (-0.04-2.12) |
| Philippines | 0.30 (0.07-0.51) | 0.47 (0.11-0.82) | 1.83 (0.41-3.17) | 1.61 (0.36-2.80) | 3.70 (2.83-4.59) |
| Guinea-Bissau | 0.01 (0.00-0.02) | 1.41 (0.34-2.39) | 0.03 (0.01-0.06) | 1.60 (0.36-2.80) | 0.74 (-0.80-2.29) |
| Ireland | 0.08 (0.02-0.13) | 2.23 (0.52-3.60) | 0.08 (0.02-0.13) | 1.56 (0.36-2.56) | -1.77 (-2.19--1.35) |
| Mali | 0.11 (0.03-0.20) | 1.30 (0.31-2.30) | 0.36 (0.09-0.62) | 1.50 (0.37-2.56) | 1.05 (-0.38-2.50) |
| Equatorial Guinea | 0.01 (0.00-0.01) | 1.29 (0.30-2.25) | 0.02 (0.01-0.04) | 1.49 (0.36-2.79) | 1.47 (-0.11-3.06) |
| Iceland | 0.00 (0.00-0.01) | 1.25 (0.30-2.04) | 0.01 (0.00-0.01) | 1.46 (0.35-2.36) | -0.19 (-0.73-0.35) |
| Liberia | 0.03 (0.01-0.06) | 1.32 (0.31-2.28) | 0.08 (0.02-0.14) | 1.46 (0.35-2.65) | 0.49 (-0.81-1.80) |
| South Sudan | 0.08 (0.02-0.14) | 1.28 (0.30-2.40) | 0.14 (0.03-0.26) | 1.46 (0.34-2.73) | 0.45 (-1.06-1.97) |
| Namibia | 0.01 (0.00-0.02) | 0.95 (0.21-1.75) | 0.03 (0.01-0.06) | 1.42 (0.35-2.60) | 0.77 (-0.38-1.93) |
| Bermuda | 0.00 (0.00-0.00) | 1.41 (0.32-2.44) | 0.00 (0.00-0.00) | 1.41 (0.30-2.55) | -1.39 (-2.00--0.77) |
| Bahamas | 0.00 (0.00-0.01) | 1.11 (0.26-1.97) | 0.01 (0.00-0.01) | 1.40 (0.33-2.47) | -0.53 (-1.02--0.03) |
| Togo | 0.02 (0.01-0.04) | 0.65 (0.16-1.13) | 0.12 (0.03-0.22) | 1.40 (0.38-2.63) | 2.10 (0.57-3.66) |
| Solomon Islands | 0.00 (0.00-0.00) | 0.70 (0.15-1.37) | 0.01 (0.00-0.02) | 1.38 (0.33-2.45) | 1.82 (0.65-2.99) |
| Egypt | 0.22 (0.05-0.39) | 0.40 (0.09-0.70) | 1.46 (0.32-2.56) | 1.38 (0.30-2.42) | 4.85 (3.70-6.02) |
| Haiti | 0.08 (0.02-0.15) | 1.29 (0.29-2.30) | 0.18 (0.04-0.34) | 1.36 (0.34-2.62) | 0.26 (-0.74-1.26) |
| Nigeria | 1.28 (0.32-2.16) | 1.42 (0.35-2.40) | 3.11 (0.81-5.29) | 1.35 (0.35-2.29) | 0.17 (-1.20-1.56) |
| Panama | 0.01 (0.00-0.02) | 0.51 (0.12-0.87) | 0.06 (0.01-0.10) | 1.33 (0.31-2.39) | 2.15 (1.71-2.59) |
| Botswana | 0.01 (0.00-0.02) | 0.83 (0.19-1.54) | 0.03 (0.01-0.06) | 1.33 (0.29-2.46) | 1.33 (0.14-2.52) |
| Belarus | 0.10 (0.02-0.16) | 0.95 (0.23-1.53) | 0.12 (0.03-0.20) | 1.32 (0.31-2.17) | -1.42 (-2.33--0.51) |

**Supplementary TABLE 1. (Continued)**

| Country/Territory | Death cases, n × 10³ (95% UI) (1990) | ASMR per 10⁵, n (95% UI) (2021) | Death cases, n × 10³ (95% UI) (2021) | ASMR per 10⁵, n (95% UI) (2021) | EAPC ASMR, n (95% CI) (1990-2021) |
| --- | --- | --- | --- | --- | --- |
| Benin | 0.05 (0.01-0.08) | 0.97 (0.25-1.66) | 0.18 (0.04-0.32) | 1.30 (0.28-2.34) | 1.18 (-0.28-2.67) |
| Central African Republic | 0.04 (0.01-0.06) | 1.31 (0.33-2.31) | 0.07 (0.02-0.13) | 1.28 (0.32-2.34) | 0.06 (-1.34-1.47) |
| Vanuatu | 0.00 (0.00-0.00) | 0.64 (0.15-1.22) | 0.00 (0.00-0.01) | 1.26 (0.31-2.19) | 1.63 (0.48-2.78) |
| Burkina Faso | 0.11 (0.02-0.20) | 1.19 (0.26-2.09) | 0.28 (0.06-0.48) | 1.24 (0.27-2.12) | 0.32 (-1.02-1.68) |
| Kazakhstan | 0.13 (0.03-0.22) | 0.82 (0.19-1.33) | 0.23 (0.06-0.40) | 1.22 (0.31-2.11) | -0.25 (-0.68-0.17) |
| Costa Rica | 0.01 (0.00-0.02) | 0.43 (0.10-0.78) | 0.06 (0.01-0.10) | 1.18 (0.29-2.05) | 1.19 (0.42-1.96) |
| Eritrea | 0.03 (0.01-0.05) | 0.85 (0.20-1.60) | 0.08 (0.02-0.15) | 1.18 (0.29-2.21) | 0.75 (-0.81-2.32) |
| Djibouti | 0.00 (0.00-0.00) | 0.49 (0.12-0.89) | 0.01 (0.00-0.03) | 1.16 (0.31-2.12) | 2.23 (0.74-3.75) |
| Dominican Republic | 0.03 (0.01-0.05) | 0.41 (0.10-0.73) | 0.13 (0.03-0.23) | 1.15 (0.26-2.08) | 2.86 (2.26-3.46) |
| Sierra Leone | 0.04 (0.01-0.07) | 0.93 (0.21-1.67) | 0.10 (0.02-0.18) | 1.15 (0.24-2.04) | 1.05 (-0.18-2.28) |
| Sri Lanka | 0.06 (0.01-0.11) | 0.36 (0.08-0.63) | 0.25 (0.05-0.46) | 1.11 (0.23-2.07) | 3.28 (2.75-3.81) |
| Japan | 1.87 (0.44-3.08) | 1.49 (0.35-2.45) | 1.40 (0.35-2.32) | 1.10 (0.27-1.82) | -2.27 (-3.40--1.13) |
| Zimbabwe | 0.05 (0.01-0.09) | 0.49 (0.10-0.88) | 0.17 (0.04-0.32) | 1.09 (0.26-2.03) | 2.96 (1.52-4.42) |
| Malawi | 0.09 (0.02-0.16) | 0.88 (0.21-1.59) | 0.20 (0.05-0.37) | 1.05 (0.23-1.92) | 0.35 (-1.10-1.83) |
| Nicaragua | 0.01 (0.00-0.02) | 0.37 (0.08-0.64) | 0.07 (0.02-0.12) | 1.04 (0.24-1.85) | 2.58 (1.61-3.56) |
| Mozambique | 0.11 (0.03-0.19) | 0.80 (0.20-1.42) | 0.32 (0.08-0.57) | 1.03 (0.24-1.84) | 1.76 (0.36-3.18) |
| Guam | 0.00 (0.00-0.00) | 0.72 (0.18-1.29) | 0.00 (0.00-0.00) | 1.03 (0.23-1.84) | -0.05 (-0.57-0.46) |
| Rwanda | 0.08 (0.02-0.15) | 1.16 (0.26-2.12) | 0.13 (0.03-0.26) | 1.01 (0.21-1.96) | -1.45 (-2.88--0.00) |
| Democratic Republic of the Congo | 0.45 (0.10-0.80) | 1.17 (0.27-2.11) | 0.90 (0.20-1.63) | 1.00 (0.22-1.81) | -0.61 (-2.01-0.82) |
| Lebanon | 0.02 (0.01-0.04) | 0.73 (0.17-1.32) | 0.06 (0.01-0.10) | 0.99 (0.25-1.82) | 0.35 (0.04-0.67) |
| Türkiye | 0.30 (0.07-0.53) | 0.53 (0.11-0.93) | 0.83 (0.20-1.46) | 0.99 (0.24-1.75) | 1.58 (0.97-2.20) |
| Tajikistan | 0.04 (0.01-0.07) | 0.76 (0.18-1.30) | 0.10 (0.02-0.18) | 0.97 (0.22-1.72) | 0.30 (-0.71-1.31) |

**Supplementary TABLE 1. (Continued)**

| Country/Territory | Death cases, n × 10³ (95% UI) (1990) | ASMR per 10⁵, n (95% UI) (2021) | Death cases, n × 10³ (95% UI) (2021) | ASMR per 10⁵, n (95% UI) (2021) | EAPC ASMR, n (95% CI) (1990-2021) |
| --- | --- | --- | --- | --- | --- |
| Chad | 0.05 (0.01-0.09) | 0.81 (0.19-1.48) | 0.17 (0.05-0.31) | 0.97 (0.26-1.76) | 1.18 (-0.29-2.68) |
| Malaysia | 0.09 (0.02-0.16) | 0.52 (0.12-0.90) | 0.31 (0.07-0.54) | 0.96 (0.21-1.69) | 1.11 (0.37-1.86) |
| Papua New Guinea | 0.03 (0.01-0.07) | 0.84 (0.20-1.59) | 0.10 (0.02-0.18) | 0.96 (0.20-1.73) | 0.23 (-0.97-1.45) |
| Kenya | 0.12 (0.03-0.21) | 0.50 (0.12-0.92) | 0.47 (0.12-0.82) | 0.94 (0.24-1.63) | 1.98 (0.49-3.48) |
| Angola | 0.09 (0.02-0.15) | 0.83 (0.22-1.51) | 0.31 (0.06-0.55) | 0.93 (0.19-1.67) | 0.51 (-1.04-2.08) |
| United Republic of Tanzania | 0.22 (0.05-0.40) | 0.86 (0.20-1.55) | 0.54 (0.12-0.98) | 0.93 (0.20-1.68) | 0.09 (-1.22-1.43) |
| Cuba | 0.10 (0.02-0.18) | 0.93 (0.22-1.63) | 0.10 (0.02-0.18) | 0.92 (0.21-1.64) | -1.00 (-1.66--0.35) |
| Ecuador | 0.04 (0.01-0.07) | 0.42 (0.09-0.73) | 0.17 (0.04-0.29) | 0.92 (0.21-1.62) | 1.75 (1.13-2.36) |
| Sao Tome and Principe | 0.00 (0.00-0.00) | 0.65 (0.15-1.12) | 0.00 (0.00-0.00) | 0.90 (0.20-1.55) | 1.16 (0.04-2.28) |
| Honduras | 0.01 (0.00-0.02) | 0.29 (0.06-0.50) | 0.09 (0.02-0.17) | 0.89 (0.20-1.70) | 3.39 (2.38-4.41) |
| Mongolia | 0.01 (0.00-0.01) | 0.38 (0.08-0.69) | 0.03 (0.01-0.05) | 0.88 (0.21-1.48) | 2.44 (1.51-3.38) |
| Zambia | 0.07 (0.02-0.12) | 0.88 (0.22-1.52) | 0.17 (0.04-0.30) | 0.85 (0.22-1.55) | -0.58 (-2.14-1.00) |
| Myanmar | 0.20 (0.05-0.36) | 0.49 (0.12-0.88) | 0.47 (0.11-0.86) | 0.84 (0.19-1.53) | 1.01 (0.39-1.63) |
| Kyrgyzstan | 0.02 (0.01-0.04) | 0.52 (0.13-0.87) | 0.06 (0.01-0.09) | 0.83 (0.20-1.37) | 0.28 (-0.42-0.99) |
| Uganda | 0.13 (0.03-0.26) | 0.75 (0.16-1.48) | 0.35 (0.07-0.65) | 0.81 (0.17-1.50) | -0.04 (-1.71-1.66) |
| Niger | 0.05 (0.01-0.09) | 0.63 (0.15-1.11) | 0.20 (0.05-0.38) | 0.81 (0.20-1.52) | 1.08 (-0.63-2.82) |
| Colombia | 0.13 (0.03-0.23) | 0.40 (0.09-0.70) | 0.39 (0.08-0.68) | 0.79 (0.16-1.38) | 0.17 (-0.38-0.72) |
| Burundi | 0.06 (0.02-0.11) | 1.14 (0.27-2.06) | 0.10 (0.02-0.19) | 0.77 (0.16-1.45) | -1.46 (-2.92-0.01) |
| Somalia | 0.06 (0.01-0.11) | 0.79 (0.17-1.42) | 0.16 (0.04-0.29) | 0.73 (0.17-1.34) | -0.08 (-1.81-1.67) |
| Thailand | 0.11 (0.03-0.21) | 0.20 (0.05-0.36) | 0.48 (0.10-0.86) | 0.71 (0.16-1.28) | 2.03 (1.58-2.48) |
| Madagascar | 0.10 (0.02-0.18) | 0.82 (0.19-1.48) | 0.20 (0.04-0.37) | 0.71 (0.16-1.28) | -0.17 (-1.65-1.33) |
| Ukraine | 0.27 (0.06-0.46) | 0.51 (0.12-0.88) | 0.30 (0.07-0.54) | 0.69 (0.16-1.24) | -0.47 (-1.14-0.20) |

**Supplementary TABLE 1. (Continued)**

| Country/Territory | Death cases, n × 10³ (95% UI) (1990) | ASMR per 10⁵, n (95% UI) (2021) | Death cases, n × 10³ (95% UI) (2021) | ASMR per 10⁵, n (95% UI) (2021) | EAPC ASMR, n (95% CI) (1990-2021) |
| --- | --- | --- | --- | --- | --- |
| Ethiopia | 0.58 (0.14-1.10) | 1.15 (0.28-2.17) | 0.75 (0.19-1.28) | 0.69 (0.17-1.18) | -1.94 (-3.41--0.45) |
| Iraq | 0.09 (0.02-0.16) | 0.47 (0.10-0.84) | 0.27 (0.06-0.50) | 0.65 (0.15-1.20) | 0.76 (-0.43-1.95) |
| Tunisia | 0.01 (0.00-0.03) | 0.18 (0.04-0.32) | 0.07 (0.02-0.14) | 0.63 (0.14-1.15) | 3.19 (2.67-3.72) |
| Bolivia (Plurinational State of) | 0.02 (0.00-0.04) | 0.34 (0.07-0.61) | 0.07 (0.02-0.13) | 0.61 (0.14-1.12) | 1.38 (0.61-2.16) |
| Morocco | 0.03 (0.01-0.05) | 0.11 (0.03-0.20) | 0.22 (0.05-0.40) | 0.60 (0.14-1.08) | 5.47 (4.76-6.19) |
| Jordan | 0.02 (0.00-0.03) | 0.47 (0.11-0.82) | 0.07 (0.01-0.14) | 0.59 (0.12-1.10) | -0.52 (-1.90-0.87) |
| Qatar | 0.00 (0.00-0.00) | 0.51 (0.12-0.91) | 0.02 (0.00-0.03) | 0.59 (0.13-1.11) | 0.08 (-2.32-2.53) |
| Democratic People's Republic of Korea | 0.06 (0.01-0.12) | 0.30 (0.07-0.58) | 0.15 (0.03-0.28) | 0.57 (0.13-1.05) | 1.46 (1.19-1.72) |
| Iran (Islamic Republic of) | 0.09 (0.02-0.16) | 0.15 (0.03-0.28) | 0.48 (0.11-0.83) | 0.56 (0.13-0.98) | 3.50 (2.58-4.43) |
| Libya | 0.01 (0.00-0.01) | 0.19 (0.04-0.33) | 0.04 (0.01-0.07) | 0.54 (0.13-0.96) | 3.38 (2.40-4.38) |
| Saudi Arabia | 0.03 (0.01-0.06) | 0.21 (0.05-0.38) | 0.20 (0.04-0.36) | 0.53 (0.11-0.96) | 2.05 (0.74-3.38) |
| China | 1.46 (0.33-2.55) | 0.12 (0.03-0.22) | 7.19 (1.60-12.71) | 0.51 (0.11-0.89) | 3.71 (3.30-4.13) |
| United Arab Emirates | 0.01 (0.00-0.02) | 0.62 (0.14-1.05) | 0.05 (0.01-0.09) | 0.51 (0.11-0.91) | -0.54 (-3.08-2.07) |
| Syrian Arab Republic | 0.03 (0.01-0.05) | 0.21 (0.05-0.37) | 0.07 (0.02-0.13) | 0.49 (0.12-0.90) | 0.99 (-0.28-2.28) |
| Palestine | 0.01 (0.00-0.02) | 0.44 (0.09-0.81) | 0.02 (0.01-0.04) | 0.48 (0.11-0.84) | 0.17 (-1.32-1.68) |
| Oman | 0.01 (0.00-0.01) | 0.30 (0.07-0.55) | 0.02 (0.00-0.04) | 0.46 (0.10-0.85) | 1.75 (0.19-3.33) |
| Kuwait | 0.00 (0.00-0.01) | 0.19 (0.05-0.34) | 0.02 (0.00-0.04) | 0.44 (0.10-0.80) | 1.14 (-0.21-2.51) |
| Seychelles | 0.00 (0.00-0.00) | 0.21 (0.04-0.36) | 0.00 (0.00-0.00) | 0.44 (0.10-0.77) | 2.32 (2.04-2.61) |
| Cambodia | 0.02 (0.00-0.04) | 0.19 (0.04-0.35) | 0.07 (0.02-0.13) | 0.40 (0.09-0.77) | 1.81 (0.82-2.80) |
| Algeria | 0.03 (0.01-0.06) | 0.12 (0.03-0.22) | 0.18 (0.04-0.33) | 0.40 (0.09-0.74) | 3.51 (2.51-4.52) |
| Lao People's Democratic Republic | 0.01 (0.00-0.02) | 0.28 (0.06-0.51) | 0.03 (0.01-0.05) | 0.39 (0.09-0.69) | 0.64 (-0.35-1.64) |
| Viet Nam | 0.11 (0.02-0.19) | 0.16 (0.03-0.28) | 0.38 (0.08-0.68) | 0.38 (0.08-0.68) | 2.42 (1.79-3.05) |

**Supplementary TABLE 1. (Continued)**

| Country/Territory | Death cases, n × 10³ (95% UI) (1990) | ASMR per 10⁵, n (95% UI) (2021) | Death cases, n × 10³ (95% UI) (2021) | ASMR per 10⁵, n (95% UI) (2021) | EAPC ASMR, n (95% CI) (1990-2021) |
| --- | --- | --- | --- | --- | --- |
| Peru | 0.02 (0.01-0.04) | 0.11 (0.02-0.20) | 0.12 (0.03-0.24) | 0.34 (0.08-0.65) | 2.85 (2.26-3.44) |
| Indonesia | 0.21 (0.05-0.37) | 0.12 (0.03-0.20) | 0.89 (0.18-1.57) | 0.32 (0.07-0.56) | 2.71 (1.99-3.44) |
| India | 1.08 (0.24-1.95) | 0.13 (0.03-0.23) | 4.38 (0.92-7.71) | 0.31 (0.07-0.54) | 2.22 (1.35-3.11) |
| Singapore | 0.02 (0.01-0.04) | 0.78 (0.18-1.33) | 0.02 (0.00-0.03) | 0.27 (0.06-0.45) | -4.56 (-5.36--3.75) |
| Afghanistan | 0.04 (0.01-0.08) | 0.42 (0.09-0.79) | 0.08 (0.02-0.17) | 0.27 (0.05-0.53) | 0.34 (-0.98-1.69) |
| Timor-Leste | 0.00 (0.00-0.00) | 0.09 (0.02-0.17) | 0.00 (0.00-0.01) | 0.21 (0.05-0.40) | 2.14 (0.99-3.29) |
| Sudan | 0.03 (0.01-0.05) | 0.13 (0.03-0.23) | 0.09 (0.02-0.16) | 0.20 (0.05-0.37) | 1.76 (0.52-3.02) |
| Maldives | 0.00 (0.00-0.00) | 0.21 (0.05-0.38) | 0.00 (0.00-0.00) | 0.18 (0.04-0.32) | -1.45 (-2.51--0.37) |
| Yemen | 0.01 (0.00-0.02) | 0.09 (0.02-0.17) | 0.04 (0.01-0.08) | 0.12 (0.03-0.24) | 1.11 (-0.38-2.63) |

**NOTE**: ASMR: Age-Standardized Mortality Rate; EAPC: Estimated Annual Percentage Change; UI: Uncertainty Interval; CI: Confidence Interval

**Supplementary Table 2** DALYs and ASDR of T2D attributable to high Intake of processed meat across 204 countries and territories

| Country/Territory | DALYs, n × 10³ (95% UI) 1990 | ASDR per 10⁵, n (95% UI) 1990 | DALYs, n × 10³ (95% UI) 2021 | ASDR per 10⁵, n (95% UI) 2021 | EAPC ASDR, n (95% CI) (1990-2021) |
| --- | --- | --- | --- | --- | --- |
| Bosnia and Herzegovina | 3.88 (0.90-6.49) | 86.26 (19.94-144.35) | 12.27 (2.93-21.39) | 371.56 (88.69-647.47) | 4.03 (3.43-4.63) |
| United States of America | 353.51 (87.68-577.95) | 139.12 (34.51-227.45) | 1048.21 (261.85-1775.63) | 315.12 (78.72-533.79) | 1.95 (1.48-2.42) |
| North Macedonia | 2.31 (0.56-3.92) | 115.97 (28.35-196.64) | 6.67 (1.54-11.41) | 306.35 (70.79-524.41) | 2.52 (2.14-2.90) |
| Bulgaria | 13.38 (3.14-23.16) | 154.16 (36.18-266.79) | 20.45 (4.80-35.35) | 301.27 (70.67-520.82) | 1.70 (0.94-2.48) |
| Malta | 0.53 (0.13-0.86) | 143.52 (35.50-231.66) | 1.26 (0.31-2.13) | 283.92 (69.58-481.30) | 1.38 (0.74-2.03) |
| Latvia | 2.35 (0.58-3.86) | 88.47 (21.96-145.22) | 5.26 (1.31-8.71) | 281.40 (70.23-465.69) | 3.00 (2.31-3.70) |
| Czechia | 12.40 (2.97-21.24) | 120.43 (28.84-206.34) | 27.87 (6.85-49.18) | 262.09 (64.43-462.51) | 2.79 (2.09-3.51) |
| Trinidad and Tobago | 1.64 (0.38-2.87) | 135.99 (31.55-237.89) | 3.59 (0.85-6.55) | 258.05 (60.67-470.42) | 0.90 (0.61-1.20) |
| Montenegro | 0.69 (0.16-1.18) | 110.21 (25.21-189.14) | 1.58 (0.39-2.70) | 255.01 (63.80-437.07) | 2.07 (1.64-2.50) |
|  |  |  |  |  |  |

**Supplementary TABLE 2. (Continued)**

| Country/Territory | DALYs, n × 10³ (95% UI) 1990 | ASDR per 10⁵, n (95% UI) 1990 | DALYs, n × 10³ (95% UI) 2021 | ASDR per 10⁵, n (95% UI) 2021 | EAPC ASDR, n (95% CI) (1990-2021) |
| --- | --- | --- | --- | --- | --- |
| Serbia | 12.43 (3.06-20.67) | 129.10 (31.77-214.64) | 22.55 (5.40-40.01) | 252.78 (60.58-448.54) | 1.44 (0.85-2.03) |
| Hungary | 13.54 (3.09-23.35) | 130.21 (29.68-224.62) | 24.22 (5.59-41.75) | 252.37 (58.27-434.98) | 1.98 (1.28-2.69) |
| Spain | 69.21 (16.60-116.54) | 178.47 (42.81-300.51) | 108.72 (25.56-190.22) | 238.70 (56.10-417.62) | 0.17 (-0.53-0.87) |
| Estonia | 1.17 (0.29-1.97) | 74.51 (18.41-125.91) | 3.07 (0.76-5.11) | 234.26 (57.87-389.79) | 2.80 (2.13-3.46) |
| Brunei Darussalam | 0.28 (0.07-0.48) | 108.38 (26.64-184.87) | 1.04 (0.26-1.85) | 231.44 (57.80-410.20) | 1.32 (0.50-2.16) |
| Slovenia | 2.51 (0.61-4.23) | 126.96 (31.10-214.53) | 4.50 (1.11-7.73) | 217.60 (53.87-373.43) | 0.18 (-0.51-0.87) |
| Lithuania | 2.29 (0.57-3.85) | 62.26 (15.40-104.71) | 5.93 (1.47-9.97) | 217.21 (53.81-365.33) | 3.04 (2.41-3.67) |
| Portugal | 11.72 (2.73-20.49) | 115.65 (26.98-202.13) | 23.04 (5.45-40.47) | 217.20 (51.37-381.49) | 1.19 (0.47-1.92) |
| Fiji | 0.61 (0.14-1.06) | 80.36 (18.69-140.18) | 1.99 (0.43-3.62) | 215.26 (46.73-391.18) | 2.30 (1.73-2.88) |
| Cook Islands | 0.02 (0.00-0.03) | 90.46 (21.02-159.68) | 0.04 (0.01-0.07) | 213.42 (47.74-378.15) | 1.61 (1.31-1.90) |
| Republic of Korea | 28.08 (6.65-48.66) | 63.47 (15.04-109.99) | 107.65 (25.85-196.14) | 208.75 (50.13-380.33) | 2.32 (1.83-2.82) |
| Russian Federation | 106.18 (26.71-175.90) | 70.32 (17.69-116.50) | 301.52 (73.79-499.13) | 208.16 (50.94-344.57) | 2.47 (1.89-3.06) |
| Cyprus | 1.78 (0.44-2.93) | 229.31 (56.99-376.31) | 2.79 (0.66-4.81) | 205.38 (48.93-354.06) | -1.44 (-1.72--1.16) |
| Georgia | 4.22 (1.08-7.15) | 76.36 (19.54-129.38) | 7.31 (1.67-12.54) | 202.53 (46.19-347.76) | 3.43 (2.90-3.96) |
| Uruguay | 3.30 (0.78-5.46) | 105.16 (24.99-174.00) | 6.90 (1.65-11.88) | 202.50 (48.51-348.83) | 1.85 (1.42-2.28) |
| Italy | 91.43 (22.60-148.96) | 160.98 (39.79-262.26) | 120.88 (29.36-201.84) | 202.09 (49.10-337.46) | 0.34 (-0.52-1.21) |
| Chile | 10.76 (2.67-17.65) | 80.97 (20.12-132.82) | 37.05 (8.71-66.34) | 197.11 (46.31-352.92) | 2.09 (1.80-2.38) |
| United States Virgin Islands | 0.07 (0.02-0.12) | 64.84 (14.37-113.94) | 0.17 (0.04-0.32) | 195.99 (42.73-369.58) | 2.44 (1.96-2.93) |
| Canada | 20.58 (5.13-34.74) | 75.52 (18.81-127.47) | 72.55 (18.12-127.36) | 193.62 (48.35-339.90) | 1.76 (1.16-2.37) |
| Japan | 121.60 (30.71-206.47) | 96.64 (24.41-164.09) | 244.72 (58.41-439.55) | 191.64 (45.74-344.21) | 1.46 (0.66-2.26) |
| Switzerland | 9.19 (2.24-15.43) | 133.85 (32.65-224.66) | 17.09 (4.10-30.09) | 191.50 (45.90-337.27) | 0.65 (-0.02-1.34) |
| Germany | 99.16 (24.43-163.31) | 124.04 (30.57-204.29) | 162.10 (39.29-273.94) | 189.87 (46.03-320.88) | 0.78 (-0.06-1.63) |

**Supplementary TABLE 2. (Continued)**

| Country/Territory | DALYs, n × 10³ (95% UI) 1990 | ASDR per 10⁵, n (95% UI) 1990 | DALYs, n × 10³ (95% UI) 2021 | ASDR per 10⁵, n (95% UI) 2021 | EAPC ASDR, n (95% CI) (1990-2021) |
| --- | --- | --- | --- | --- | --- |
| Republic of Moldova | 3.32 (0.80-5.66) | 74.76 (18.09-127.25) | 6.80 (1.68-11.77) | 189.26 (46.84-327.56) | 1.87 (1.45-2.29) |
| Poland | 30.08 (7.68-52.19) | 78.80 (20.13-136.72) | 71.09 (17.68-121.99) | 185.91 (46.25-319.03) | 2.65 (2.11-3.21) |
| Puerto Rico | 2.83 (0.71-4.95) | 78.29 (19.64-137.02) | 6.05 (1.38-10.78) | 183.51 (41.96-327.23) | 1.57 (1.09-2.06) |
| United Kingdom | 59.45 (15.01-97.42) | 103.75 (26.19-170.03) | 122.30 (30.42-209.64) | 180.25 (44.84-308.98) | 1.54 (0.95-2.14) |
| Niue | 0.00 (0.00-0.00) | 69.38 (15.90-122.99) | 0.00 (0.00-0.01) | 179.48 (41.97-329.47) | 2.53 (2.25-2.80) |
| Palau | 0.01 (0.00-0.01) | 55.22 (13.05-97.54) | 0.03 (0.01-0.06) | 171.75 (39.57-304.61) | 2.73 (2.42-3.04) |
| Slovakia | 4.81 (1.18-8.15) | 91.08 (22.26-154.31) | 9.29 (2.24-16.10) | 171.10 (41.21-296.53) | 1.58 (1.12-2.05) |
| Barbados | 0.29 (0.06-0.52) | 115.57 (25.04-206.47) | 0.51 (0.11-0.92) | 170.27 (38.25-306.07) | 0.41 (0.01-0.82) |
| Greece | 8.74 (2.08-15.23) | 84.14 (20.06-146.57) | 17.26 (4.15-30.28) | 169.66 (40.80-297.58) | 1.71 (1.03-2.38) |
| Finland | 4.20 (1.03-7.07) | 83.80 (20.61-141.08) | 9.37 (2.21-16.21) | 169.29 (39.97-292.83) | 1.77 (1.11-2.44) |
| Saint Vincent and the Grenadines | 0.08 (0.02-0.14) | 73.30 (17.13-127.24) | 0.19 (0.05-0.35) | 167.19 (39.75-309.07) | 1.49 (1.13-1.85) |
| Guyana | 0.51 (0.12-0.88) | 65.77 (15.59-113.44) | 1.28 (0.28-2.29) | 166.78 (36.73-299.13) | 2.07 (1.45-2.69) |
| Belgium | 11.09 (2.71-18.52) | 111.16 (27.14-185.57) | 18.93 (4.57-33.28) | 165.07 (39.87-290.20) | 0.84 (0.16-1.53) |
| Sweden | 9.79 (2.38-16.35) | 113.97 (27.67-190.44) | 16.92 (4.20-28.40) | 163.07 (40.48-273.78) | 1.11 (0.34-1.88) |
| Mexico | 66.95 (16.04-113.90) | 78.40 (18.78-133.38) | 210.63 (53.38-371.65) | 162.93 (41.29-287.49) | 1.21 (0.69-1.72) |
| Dominica | 0.06 (0.01-0.10) | 77.77 (17.66-136.78) | 0.11 (0.03-0.20) | 162.68 (38.04-297.40) | 1.47 (1.28-1.67) |
| Romania | 15.43 (3.64-27.59) | 65.99 (15.58-118.02) | 30.56 (7.30-54.37) | 161.34 (38.54-287.07) | 2.63 (2.04-3.23) |
| Andorra | 0.04 (0.01-0.07) | 78.52 (19.30-133.65) | 0.14 (0.03-0.23) | 158.53 (39.60-269.43) | 1.53 (1.05-2.02) |
| American Samoa | 0.02 (0.01-0.04) | 50.86 (11.00-88.64) | 0.08 (0.02-0.14) | 157.59 (36.26-283.37) | 2.60 (2.02-3.18) |
| Denmark | 4.46 (1.08-7.48) | 86.68 (21.05-145.44) | 8.78 (2.15-15.06) | 150.04 (36.82-257.33) | 1.43 (0.71-2.15) |
| Uzbekistan | 7.75 (1.83-13.37) | 36.98 (8.74-63.79) | 51.00 (11.97-86.39) | 148.98 (34.96-252.36) | 3.75 (3.11-4.39) |
| Monaco | 0.02 (0.01-0.04) | 77.85 (19.76-131.56) | 0.06 (0.01-0.10) | 147.45 (35.40-254.64) | 2.04 (1.05-3.03) |

**Supplementary TABLE 2. (Continued)**

| Country/Territory | DALYs, n × 10³ (95% UI) 1990 | ASDR per 10⁵, n (95% UI) 1990 | DALYs, n × 10³ (95% UI) 2021 | ASDR per 10⁵, n (95% UI) 2021 | EAPC ASDR, n (95% CI) (1990-2021) |
| --- | --- | --- | --- | --- | --- |
| Taiwan (Province of China) | 9.86 (2.25-17.51) | 48.34 (11.03-85.86) | 34.34 (7.91-61.68) | 145.28 (33.48-260.96) | 1.88 (1.45-2.31) |
| Netherlands | 17.58 (4.21-29.34) | 117.82 (28.19-196.61) | 24.79 (5.95-42.42) | 144.03 (34.56-246.45) | 0.00 (-0.62-0.63) |
| San Marino | 0.02 (0.00-0.03) | 78.37 (19.13-130.71) | 0.05 (0.01-0.08) | 142.54 (35.51-249.40) | 1.62 (0.87-2.37) |
| Azerbaijan | 3.49 (0.85-5.76) | 47.61 (11.65-78.60) | 14.88 (3.42-25.25) | 141.70 (32.54-240.51) | 2.80 (2.46-3.15) |
| Saint Lucia | 0.10 (0.02-0.19) | 75.95 (17.47-137.63) | 0.25 (0.06-0.47) | 140.44 (32.99-264.20) | 0.28 (-0.11-0.68) |
| New Zealand | 3.06 (0.72-5.12) | 89.61 (20.97-149.72) | 7.26 (1.75-12.38) | 140.43 (33.83-239.48) | 0.78 (0.36-1.20) |
| Argentina | 27.77 (6.62-47.36) | 83.88 (20.00-143.03) | 63.77 (15.23-111.01) | 140.19 (33.49-244.04) | 1.23 (1.09-1.38) |
| Norway | 4.60 (1.16-7.65) | 108.29 (27.41-180.26) | 7.39 (1.87-12.57) | 136.35 (34.48-232.02) | 0.33 (-0.26-0.92) |
| Albania | 1.35 (0.33-2.31) | 40.74 (9.98-69.82) | 3.63 (0.86-6.24) | 136.23 (32.16-233.86) | 2.64 (2.23-3.06) |
| Australia | 12.83 (3.08-21.52) | 76.08 (18.25-127.65) | 35.11 (8.53-61.78) | 136.12 (33.07-239.52) | 1.15 (0.66-1.65) |
| Israel | 4.34 (1.03-7.32) | 87.38 (20.68-147.56) | 13.03 (3.14-22.28) | 135.78 (32.77-232.23) | 0.71 (0.33-1.10) |
| Grenada | 0.07 (0.02-0.12) | 75.05 (17.26-132.36) | 0.14 (0.03-0.25) | 134.67 (30.93-245.49) | 1.32 (1.02-1.63) |
| Austria | 7.64 (1.87-12.44) | 98.29 (24.01-160.08) | 12.09 (2.93-20.83) | 134.63 (32.61-231.90) | 1.15 (0.36-1.94) |
| Luxembourg | 0.40 (0.10-0.66) | 104.87 (26.49-172.57) | 0.87 (0.21-1.50) | 134.62 (33.07-233.09) | 0.63 (0.11-1.15) |
| Marshall Islands | 0.02 (0.00-0.03) | 36.33 (8.16-66.06) | 0.07 (0.02-0.14) | 132.40 (30.72-252.59) | 3.31 (2.35-4.27) |
| Turkmenistan | 1.25 (0.31-2.11) | 33.84 (8.48-57.15) | 6.80 (1.62-11.29) | 131.88 (31.47-218.84) | 3.40 (2.74-4.06) |
| Nepal | 8.70 (2.07-15.26) | 44.69 (10.64-78.36) | 40.42 (9.47-70.18) | 129.84 (30.42-225.44) | 3.03 (2.31-3.75) |
| France | 41.80 (10.17-69.60) | 72.36 (17.60-120.48) | 83.29 (19.99-144.86) | 125.45 (30.11-218.20) | 1.39 (0.66-2.12) |
| Greenland | 0.03 (0.01-0.04) | 50.63 (12.48-80.69) | 0.07 (0.02-0.12) | 124.86 (29.92-210.56) | 1.73 (1.38-2.08) |
| Suriname | 0.19 (0.04-0.33) | 48.21 (11.61-84.11) | 0.72 (0.17-1.31) | 124.31 (30.11-226.60) | 2.59 (2.28-2.91) |
| Venezuela (Bolivarian Republic of) | 6.88 (1.52-12.10) | 36.58 (8.07-64.32) | 32.92 (7.19-58.11) | 123.64 (27.00-218.24) | 2.94 (2.35-3.53) |
| Kazakhstan | 8.92 (2.06-15.64) | 54.42 (12.59-95.42) | 23.21 (5.60-41.00) | 122.42 (29.55-216.31) | 1.74 (1.53-1.95) |

**Supplementary TABLE 2. (Continued)**

| Country/Territory | DALYs, n × 10³ (95% UI) 1990 | ASDR per 10⁵, n (95% UI) 1990 | DALYs, n × 10³ (95% UI) 2021 | ASDR per 10⁵, n (95% UI) 2021 | EAPC ASDR, n (95% CI) (1990-2021) |
| --- | --- | --- | --- | --- | --- |
| Bangladesh | 41.55 (9.37-72.50) | 38.08 (8.59-66.44) | 198.27 (47.30-337.98) | 120.43 (28.73-205.29) | 2.92 (2.06-3.78) |
| Cabo Verde | 0.11 (0.02-0.19) | 30.38 (6.97-52.88) | 0.67 (0.16-1.14) | 119.40 (29.24-203.55) | 3.88 (3.27-4.49) |
| South Africa | 14.07 (3.34-24.58) | 38.00 (9.02-66.41) | 67.78 (15.95-116.88) | 119.21 (28.05-205.58) | 3.72 (3.12-4.33) |
| Armenia | 1.98 (0.49-3.49) | 58.03 (14.24-102.07) | 3.56 (0.86-6.34) | 118.90 (28.56-211.77) | 1.37 (0.84-1.90) |
| Mauritius | 0.29 (0.07-0.51) | 26.31 (6.03-46.09) | 1.51 (0.34-2.66) | 118.45 (26.80-209.08) | 5.00 (4.43-5.58) |
| Bhutan | 0.23 (0.05-0.41) | 36.72 (8.52-64.38) | 0.89 (0.22-1.59) | 117.67 (28.75-210.22) | 2.65 (1.90-3.39) |
| Pakistan | 61.14 (15.23-105.60) | 55.02 (13.71-95.03) | 271.06 (66.55-463.54) | 115.07 (28.25-196.79) | 2.09 (1.20-2.98) |
| Jamaica | 1.56 (0.37-2.81) | 66.15 (15.55-118.83) | 3.22 (0.75-5.66) | 114.90 (26.94-202.20) | 0.75 (0.45-1.05) |
| Tokelau | 0.00 (0.00-0.00) | 55.74 (12.62-101.83) | 0.00 (0.00-0.00) | 113.34 (25.01-212.80) | 1.97 (1.79-2.14) |
| Kiribati | 0.04 (0.01-0.08) | 58.87 (13.59-103.72) | 0.14 (0.03-0.25) | 113.24 (26.32-208.08) | 1.81 (1.08-2.54) |
| Singapore | 1.44 (0.34-2.60) | 47.13 (11.20-85.29) | 6.44 (1.51-12.01) | 112.47 (26.42-209.65) | 2.11 (1.81-2.41) |
| Iceland | 0.13 (0.03-0.21) | 50.43 (12.35-84.61) | 0.39 (0.09-0.68) | 111.24 (26.51-194.10) | 2.09 (1.74-2.44) |
| Saint Kitts and Nevis | 0.03 (0.01-0.06) | 80.44 (17.83-141.70) | 0.06 (0.01-0.12) | 109.60 (25.56-206.41) | 0.59 (0.27-0.90) |
| Gabon | 0.69 (0.16-1.22) | 70.28 (16.49-124.22) | 1.97 (0.47-3.52) | 108.74 (26.05-193.84) | 1.21 (0.46-1.97) |
| Ghana | 5.68 (1.40-9.56) | 37.93 (9.38-63.89) | 35.70 (8.58-60.66) | 104.24 (25.05-177.12) | 3.44 (2.44-4.45) |
| Antigua and Barbuda | 0.04 (0.01-0.06) | 60.33 (14.87-105.97) | 0.09 (0.02-0.16) | 102.83 (24.86-182.97) | 0.98 (0.73-1.23) |
| Nauru | 0.01 (0.00-0.01) | 60.98 (12.64-106.59) | 0.01 (0.00-0.02) | 101.22 (22.99-184.92) | 1.28 (0.51-2.05) |
| Belarus | 5.74 (1.39-9.79) | 54.98 (13.30-93.74) | 9.22 (2.22-15.85) | 98.90 (23.76-170.02) | 0.66 (0.12-1.20) |
| Northern Mariana Islands | 0.02 (0.00-0.03) | 36.70 (8.87-63.55) | 0.05 (0.01-0.09) | 98.05 (23.71-175.93) | 2.15 (1.52-2.79) |
| Senegal | 3.71 (0.88-6.26) | 48.55 (11.48-81.99) | 15.55 (3.60-26.59) | 98.04 (22.71-167.69) | 2.23 (1.22-3.26) |
| Brazil | 54.03 (12.48-93.23) | 36.38 (8.41-62.78) | 215.46 (49.73-382.02) | 97.78 (22.57-173.36) | 2.66 (2.30-3.02) |
| Ireland | 2.59 (0.61-4.35) | 71.98 (16.95-120.87) | 4.79 (1.12-8.38) | 97.03 (22.65-169.65) | 0.48 (0.15-0.81) |

**Supplementary TABLE 2. (Continued)**

| Country/Territory | DALYs, n × 10³ (95% UI) 1990 | ASDR per 10⁵, n (95% UI) 1990 | DALYs, n × 10³ (95% UI) 2021 | ASDR per 10⁵, n (95% UI) 2021 | EAPC ASDR, n (95% CI) (1990-2021) |
| --- | --- | --- | --- | --- | --- |
| Guatemala | 1.39 (0.33-2.40) | 16.57 (3.90-28.66) | 15.25 (3.22-27.19) | 96.69 (20.45-172.47) | 4.53 (3.64-5.42) |
| Micronesia (Federated States of) | 0.03 (0.01-0.06) | 33.63 (7.22-59.89) | 0.09 (0.02-0.17) | 91.44 (19.41-163.95) | 2.60 (1.85-3.35) |
| Lesotho | 0.46 (0.10-0.81) | 29.84 (6.83-52.86) | 1.66 (0.40-3.01) | 88.36 (21.33-160.49) | 4.32 (3.48-5.17) |
| Ukraine | 19.51 (4.45-33.94) | 37.00 (8.44-64.39) | 37.66 (8.91-67.17) | 87.41 (20.68-155.93) | 2.33 (1.79-2.87) |
| Bahrain | 0.17 (0.04-0.31) | 33.83 (7.25-60.32) | 1.34 (0.30-2.38) | 87.40 (19.55-155.31) | 1.68 (0.53-2.85) |
| Samoa | 0.06 (0.01-0.11) | 37.32 (8.43-66.28) | 0.18 (0.04-0.33) | 86.54 (20.62-153.89) | 2.39 (1.74-3.04) |
| Tonga | 0.04 (0.01-0.08) | 43.52 (10.16-77.65) | 0.09 (0.02-0.17) | 86.46 (19.18-158.65) | 1.75 (1.20-2.31) |
| El Salvador | 1.24 (0.31-2.21) | 23.41 (5.88-41.69) | 5.55 (1.34-9.85) | 85.97 (20.72-152.66) | 3.38 (2.90-3.87) |
| Tuvalu | 0.00 (0.00-0.01) | 49.29 (11.44-89.20) | 0.01 (0.00-0.02) | 84.86 (18.75-152.39) | 1.46 (1.19-1.73) |
| Bahamas | 0.11 (0.03-0.20) | 44.56 (10.32-78.46) | 0.32 (0.07-0.59) | 83.43 (19.00-152.74) | 0.92 (0.58-1.26) |
| Mali | 4.91 (1.15-8.58) | 56.70 (13.22-99.07) | 19.93 (5.16-34.35) | 82.67 (21.43-142.53) | 1.65 (0.54-2.78) |
| Paraguay | 1.00 (0.22-1.73) | 24.65 (5.43-42.68) | 5.92 (1.33-10.62) | 82.60 (18.54-148.19) | 3.58 (2.98-4.18) |
| Eswatini | 0.27 (0.06-0.48) | 33.74 (7.97-59.16) | 0.93 (0.22-1.68) | 80.72 (19.44-145.37) | 2.74 (1.47-4.03) |
| Cameroon | 5.17 (1.21-8.71) | 49.50 (11.63-83.41) | 24.95 (6.21-44.09) | 78.51 (19.54-138.73) | 1.46 (0.31-2.62) |
| Costa Rica | 0.77 (0.19-1.38) | 25.28 (6.23-45.39) | 3.70 (0.91-6.50) | 77.89 (19.14-136.94) | 2.43 (2.02-2.85) |
| Belize | 0.06 (0.01-0.11) | 31.75 (7.07-56.30) | 0.33 (0.08-0.59) | 77.61 (17.90-137.67) | 2.18 (1.38-2.98) |
| Côte d'Ivoire | 4.80 (1.08-8.17) | 39.32 (8.82-66.99) | 21.46 (5.36-36.38) | 77.03 (19.24-130.57) | 1.75 (0.50-3.02) |
| Cuba | 4.30 (1.06-7.81) | 39.61 (9.73-71.98) | 8.67 (2.08-15.86) | 76.97 (18.45-140.77) | 1.41 (0.98-1.84) |
| Congo | 1.29 (0.29-2.29) | 53.61 (11.98-95.15) | 3.99 (1.05-7.10) | 74.07 (19.55-131.64) | 0.52 (-0.45-1.49) |
| Guinea-Bissau | 0.53 (0.13-0.90) | 52.59 (12.96-89.13) | 1.52 (0.37-2.61) | 73.60 (17.85-126.70) | 1.15 (-0.07-2.39) |
| Bermuda | 0.03 (0.01-0.05) | 47.40 (11.26-83.18) | 0.05 (0.01-0.09) | 73.01 (15.57-136.96) | 0.47 (-0.09-1.02) |
| Dominican Republic | 1.48 (0.37-2.63) | 20.76 (5.13-36.79) | 7.86 (1.81-14.65) | 71.33 (16.44-133.01) | 3.63 (3.15-4.11) |

**Supplementary TABLE 2. (Continued)**

| Country/Territory | DALYs, n × 10³ (95% UI) 1990 | ASDR per 10⁵, n (95% UI) 1990 | DALYs, n × 10³ (95% UI) 2021 | ASDR per 10⁵, n (95% UI) 2021 | EAPC ASDR, n (95% CI) (1990-2021) |
| --- | --- | --- | --- | --- | --- |
| Gambia | 0.33 (0.08-0.56) | 33.18 (8.35-57.22) | 1.69 (0.40-2.94) | 70.65 (16.70-122.86) | 2.17 (0.98-3.37) |
| Liberia | 1.15 (0.29-1.97) | 46.74 (11.86-79.87) | 3.83 (0.97-6.80) | 70.12 (17.72-124.54) | 1.27 (0.23-2.33) |
| Croatia | 1.47 (0.34-2.71) | 30.22 (7.02-55.70) | 2.94 (0.64-5.42) | 69.74 (15.11-128.88) | 1.84 (1.11-2.58) |
| Mongolia | 0.46 (0.11-0.80) | 21.48 (4.93-37.10) | 2.32 (0.56-4.01) | 69.56 (16.75-120.18) | 3.40 (2.70-4.11) |
| Kyrgyzstan | 1.41 (0.34-2.48) | 31.67 (7.60-55.61) | 4.64 (1.09-8.20) | 67.65 (15.89-119.53) | 1.77 (1.24-2.29) |
| Mauritania | 0.97 (0.23-1.63) | 47.09 (11.42-79.09) | 2.95 (0.68-5.18) | 67.12 (15.41-117.81) | 0.92 (-0.06-1.91) |
| Benin | 1.76 (0.45-2.98) | 36.34 (9.31-61.44) | 9.06 (2.14-15.45) | 67.11 (15.82-114.44) | 1.88 (0.67-3.10) |
| Panama | 0.56 (0.13-0.99) | 23.52 (5.48-41.27) | 2.87 (0.71-5.18) | 66.95 (16.58-120.80) | 2.68 (2.34-3.03) |
| Equatorial Guinea | 0.19 (0.04-0.34) | 45.37 (10.62-79.33) | 1.01 (0.24-1.83) | 66.89 (16.08-120.80) | 2.01 (0.74-3.30) |
| Guam | 0.04 (0.01-0.07) | 30.39 (7.06-53.87) | 0.11 (0.03-0.19) | 66.08 (16.11-122.40) | 1.59 (1.26-1.93) |
| Haiti | 3.11 (0.68-5.41) | 48.76 (10.64-84.83) | 8.47 (2.00-15.44) | 65.83 (15.56-120.03) | 0.94 (0.17-1.72) |
| Egypt | 8.08 (1.83-14.12) | 14.60 (3.31-25.52) | 69.05 (16.14-123.01) | 65.37 (15.28-116.45) | 5.40 (4.59-6.22) |
| Comoros | 0.18 (0.04-0.31) | 37.93 (7.93-66.62) | 0.49 (0.11-0.88) | 65.27 (14.18-117.72) | 0.92 (0.09-1.77) |
| Philippines | 12.59 (2.98-21.77) | 19.99 (4.73-34.55) | 73.52 (16.52-129.68) | 64.92 (14.59-114.51) | 3.62 (2.93-4.32) |
| Guinea | 2.94 (0.68-5.14) | 49.03 (11.29-85.73) | 8.65 (2.16-15.12) | 64.43 (16.11-112.58) | 1.30 (0.37-2.24) |
| Malaysia | 3.95 (0.90-6.92) | 22.38 (5.12-39.16) | 20.16 (4.57-36.77) | 63.38 (14.38-115.59) | 2.74 (2.19-3.30) |
| Sao Tome and Principe | 0.04 (0.01-0.06) | 30.50 (7.00-51.61) | 0.14 (0.03-0.24) | 62.49 (14.84-112.23) | 2.12 (1.20-3.05) |
| Central African Republic | 1.36 (0.35-2.37) | 49.65 (12.68-86.80) | 3.40 (0.87-6.01) | 62.08 (15.78-109.60) | 0.72 (-0.36-1.81) |
| Nicaragua | 0.74 (0.17-1.32) | 18.99 (4.41-34.00) | 4.12 (0.95-7.62) | 61.76 (14.22-114.28) | 3.08 (2.27-3.89) |
| Tajikistan | 1.73 (0.43-2.95) | 32.25 (8.02-54.97) | 6.14 (1.46-10.95) | 60.44 (14.33-107.76) | 1.49 (0.64-2.35) |
| Colombia | 6.79 (1.49-12.23) | 20.89 (4.57-37.64) | 29.50 (6.39-53.91) | 60.14 (13.03-109.88) | 2.07 (1.66-2.48) |
| Ecuador | 2.07 (0.50-3.70) | 20.73 (4.97-37.11) | 10.85 (2.49-19.11) | 60.04 (13.81-105.76) | 2.72 (2.25-3.20) |

**Supplementary TABLE 2. (Continued)**

| Country/Territory | DALYs, n × 10³ (95% UI) 1990 | ASDR per 10⁵, n (95% UI) 1990 | DALYs, n × 10³ (95% UI) 2021 | ASDR per 10⁵, n (95% UI) 2021 | EAPC ASDR, n (95% CI) (1990-2021) |
| --- | --- | --- | --- | --- | --- |
| Kuwait | 0.27 (0.06-0.50) | 15.45 (3.60-29.00) | 2.75 (0.59-5.21) | 59.08 (12.63-112.08) | 3.15 (2.40-3.91) |
| Togo | 0.89 (0.21-1.56) | 24.44 (5.80-42.76) | 4.94 (1.28-8.78) | 58.98 (15.26-104.84) | 2.36 (1.16-3.57) |
| Lebanon | 0.90 (0.21-1.63) | 30.11 (7.08-54.52) | 3.11 (0.72-5.73) | 56.22 (12.95-103.40) | 1.57 (1.37-1.77) |
| Solomon Islands | 0.09 (0.02-0.17) | 25.50 (5.47-50.03) | 0.38 (0.09-0.68) | 56.21 (13.54-99.29) | 2.19 (1.28-3.10) |
| Vanuatu | 0.04 (0.01-0.07) | 23.99 (5.74-44.54) | 0.17 (0.04-0.30) | 55.47 (13.17-94.60) | 2.14 (1.27-3.02) |
| Burkina Faso | 3.83 (0.84-6.72) | 40.20 (8.78-70.55) | 12.60 (2.87-21.63) | 55.35 (12.62-95.01) | 1.12 (0.02-2.24) |
| Honduras | 0.85 (0.19-1.51) | 17.97 (3.99-32.09) | 5.57 (1.24-10.33) | 55.12 (12.26-102.20) | 3.25 (2.44-4.06) |
| Sierra Leone | 1.41 (0.32-2.55) | 34.08 (7.83-61.34) | 4.86 (1.15-8.69) | 54.86 (13.02-98.01) | 1.72 (0.71-2.73) |
| Qatar | 0.11 (0.02-0.19) | 23.92 (5.46-43.46) | 1.61 (0.37-3.06) | 54.03 (12.47-102.81) | 1.66 (0.20-3.14) |
| United Arab Emirates | 0.56 (0.13-0.96) | 30.08 (7.06-51.06) | 5.14 (1.15-9.43) | 53.41 (11.97-97.91) | 0.23 (-1.35-1.83) |
| Nigeria | 41.85 (10.13-70.68) | 46.48 (11.25-78.49) | 121.72 (30.64-208.21) | 52.66 (13.26-90.07) | 0.57 (-0.55-1.69) |
| South Sudan | 2.28 (0.55-4.12) | 38.76 (9.32-70.07) | 5.04 (1.18-9.41) | 52.12 (12.15-97.33) | 0.86 (-0.42-2.16) |
| Tunisia | 0.93 (0.21-1.71) | 11.13 (2.47-20.53) | 6.10 (1.39-11.36) | 51.50 (11.71-95.92) | 4.06 (3.68-4.44) |
| Sri Lanka | 2.33 (0.51-4.20) | 13.62 (3.00-24.49) | 11.38 (2.66-20.91) | 51.10 (11.94-93.91) | 3.75 (3.38-4.13) |
| Morocco | 1.82 (0.40-3.22) | 7.17 (1.59-12.72) | 18.99 (4.31-34.65) | 51.08 (11.59-93.19) | 6.34 (5.80-6.88) |
| Türkiye | 9.82 (2.18-17.11) | 17.08 (3.78-29.78) | 41.92 (9.74-76.08) | 50.14 (11.64-90.99) | 2.99 (2.48-3.51) |
| Eritrea | 1.10 (0.25-2.00) | 32.22 (7.40-58.61) | 3.27 (0.78-5.94) | 49.49 (11.81-90.05) | 1.00 (-0.17-2.19) |
| Botswana | 0.34 (0.08-0.62) | 25.44 (6.05-46.65) | 1.15 (0.26-2.06) | 48.23 (11.07-86.13) | 1.70 (0.72-2.68) |
| Angola | 3.36 (0.86-6.06) | 32.66 (8.40-58.99) | 15.11 (3.56-27.18) | 46.20 (10.88-83.11) | 1.24 (0.04-2.46) |
| Namibia | 0.40 (0.09-0.73) | 28.66 (6.21-51.85) | 1.12 (0.28-2.02) | 46.15 (11.33-83.05) | 1.00 (0.02-1.98) |
| Djibouti | 0.07 (0.02-0.13) | 17.75 (4.40-32.58) | 0.58 (0.14-1.02) | 45.77 (11.20-81.09) | 2.47 (1.37-3.59) |
| Papua New Guinea | 1.30 (0.31-2.44) | 31.72 (7.49-59.40) | 4.78 (1.02-8.50) | 45.70 (9.79-81.22) | 0.92 (0.02-1.82) |

**Supplementary TABLE 2. (Continued)**

| Country/Territory | DALYs, n × 10³ (95% UI) 1990 | ASDR per 10⁵, n (95% UI) 1990 | DALYs, n × 10³ (95% UI) 2021 | ASDR per 10⁵, n (95% UI) 2021 | EAPC ASDR, n (95% CI) (1990-2021) |
| --- | --- | --- | --- | --- | --- |
| Chad | 1.80 (0.43-3.20) | 29.92 (7.21-53.18) | 7.93 (2.07-13.78) | 44.69 (11.67-77.65) | 1.67 (0.42-2.94) |
| Democratic Republic of the Congo | 15.74 (3.90-27.69) | 41.27 (10.21-72.57) | 38.75 (8.63-68.02) | 43.05 (9.59-75.56) | -0.03 (-1.16-1.11) |
| Iraq | 3.77 (0.91-6.83) | 20.47 (4.97-37.08) | 17.73 (4.13-32.04) | 43.01 (10.02-77.72) | 2.08 (1.15-3.01) |
| Saudi Arabia | 1.72 (0.37-3.00) | 10.86 (2.35-18.93) | 16.15 (3.69-29.09) | 42.83 (9.79-77.15) | 3.49 (2.60-4.39) |
| Jordan | 0.71 (0.16-1.27) | 19.00 (4.23-33.91) | 5.24 (1.14-9.63) | 42.55 (9.28-78.17) | 1.49 (0.45-2.54) |
| Niger | 2.19 (0.52-3.70) | 27.27 (6.45-46.01) | 10.52 (2.66-18.85) | 42.02 (10.63-75.29) | 1.56 (0.21-2.93) |
| Libya | 0.45 (0.10-0.81) | 10.59 (2.43-19.10) | 2.86 (0.68-5.13) | 41.61 (9.87-74.60) | 3.97 (3.21-4.74) |
| China | 89.90 (20.29-159.47) | 7.64 (1.72-13.56) | 580.12 (135.85-1043.11) | 40.77 (9.55-73.32) | 5.11 (4.79-5.44) |
| Mozambique | 3.53 (0.90-6.29) | 26.44 (6.76-47.10) | 12.52 (2.92-21.64) | 40.30 (9.41-69.65) | 2.21 (1.05-3.38) |
| Zimbabwe | 1.63 (0.34-2.87) | 15.79 (3.33-27.72) | 6.03 (1.47-10.81) | 38.66 (9.40-69.30) | 3.22 (1.99-4.48) |
| Zambia | 2.45 (0.63-4.37) | 30.90 (8.00-55.01) | 7.48 (1.82-13.25) | 38.33 (9.33-67.90) | 0.11 (-1.18-1.42) |
| Algeria | 2.04 (0.47-3.58) | 8.08 (1.84-14.14) | 16.01 (3.77-30.05) | 36.22 (8.53-67.99) | 4.36 (3.71-5.01) |
| Syrian Arab Republic | 1.33 (0.30-2.37) | 10.42 (2.39-18.60) | 5.04 (1.18-9.36) | 35.89 (8.42-66.75) | 2.47 (1.53-3.41) |
| Malawi | 2.72 (0.66-4.96) | 27.72 (6.68-50.63) | 6.96 (1.53-12.53) | 35.81 (7.87-64.41) | 0.55 (-0.69-1.80) |
| Rwanda | 2.69 (0.60-4.90) | 37.45 (8.40-68.17) | 4.66 (0.97-8.52) | 35.09 (7.28-64.24) | -1.32 (-2.50--0.14) |
| Iran (Islamic Republic of) | 4.58 (1.04-8.21) | 8.03 (1.82-14.37) | 29.88 (7.03-52.64) | 35.01 (8.24-61.67) | 3.93 (3.28-4.58) |
| Democratic People's Republic of Korea | 3.01 (0.67-5.63) | 14.61 (3.28-27.35) | 9.05 (2.13-16.45) | 34.30 (8.06-62.34) | 2.19 (2.01-2.37) |
| Myanmar | 6.84 (1.61-12.38) | 16.91 (3.97-30.61) | 19.27 (4.30-34.40) | 34.16 (7.63-60.97) | 1.55 (1.09-2.02) |
| United Republic of Tanzania | 6.92 (1.64-12.39) | 26.79 (6.33-47.97) | 19.71 (4.33-35.57) | 33.73 (7.41-60.86) | 0.54 (-0.56-1.66) |
| Kenya | 3.73 (0.91-6.69) | 16.09 (3.92-28.90) | 16.50 (4.32-28.69) | 32.96 (8.62-57.31) | 2.11 (0.87-3.36) |
| Thailand | 4.28 (0.95-7.63) | 7.54 (1.67-13.45) | 21.85 (4.81-40.51) | 32.77 (7.21-60.75) | 3.15 (2.77-3.53) |
| Seychelles | 0.01 (0.00-0.01) | 8.96 (1.90-15.99) | 0.03 (0.01-0.06) | 31.87 (6.84-57.58) | 3.67 (3.44-3.90) |

**Supplementary TABLE 2. (Continued)**

| Country/Territory | DALYs, n × 10³ (95% UI) 1990 | ASDR per 10⁵, n (95% UI) 1990 | DALYs, n × 10³ (95% UI) 2021 | ASDR per 10⁵, n (95% UI) 2021 | EAPC ASDR, n (95% CI) (1990-2021) |
| --- | --- | --- | --- | --- | --- |
| Madagascar | 3.26 (0.72-5.75) | 27.36 (6.09-48.29) | 8.47 (1.96-14.85) | 29.66 (6.85-51.99) | 0.36 (-0.81-1.55) |
| Burundi | 1.99 (0.47-3.53) | 35.77 (8.48-63.63) | 3.90 (0.86-7.07) | 29.48 (6.52-53.47) | -1.02 (-2.23-0.19) |
| Uganda | 4.01 (0.88-7.73) | 23.17 (5.10-44.72) | 12.63 (2.74-22.87) | 29.16 (6.32-52.79) | 0.30 (-1.12-1.74) |
| Somalia | 2.26 (0.51-3.98) | 28.43 (6.45-50.09) | 6.26 (1.47-11.38) | 28.98 (6.81-52.68) | 0.13 (-1.25-1.53) |
| Oman | 0.23 (0.05-0.41) | 11.75 (2.61-20.90) | 1.30 (0.32-2.37) | 27.60 (6.76-50.33) | 2.26 (1.06-3.47) |
| Ethiopia | 20.00 (4.96-37.36) | 39.56 (9.82-73.89) | 30.01 (7.00-51.29) | 27.55 (6.42-47.08) | -1.55 (-2.75--0.33) |
| Bolivia (Plurinational State of) | 0.73 (0.15-1.30) | 11.52 (2.40-20.42) | 2.92 (0.65-5.44) | 24.72 (5.47-46.08) | 1.90 (1.25-2.55) |
| Palestine | 0.28 (0.06-0.51) | 13.75 (3.02-24.72) | 1.17 (0.27-2.11) | 22.87 (5.25-41.00) | 1.27 (0.07-2.48) |
| Afghanistan | 1.90 (0.43-3.47) | 19.15 (4.31-34.87) | 6.40 (1.40-12.09) | 20.50 (4.48-38.72) | 1.51 (0.37-2.66) |
| Lao People's Democratic Republic | 0.42 (0.09-0.75) | 9.98 (2.17-18.08) | 1.31 (0.29-2.29) | 17.82 (3.95-31.07) | 1.44 (0.61-2.28) |
| Cambodia | 0.68 (0.15-1.24) | 6.65 (1.50-12.06) | 2.93 (0.65-5.32) | 17.21 (3.83-31.19) | 2.49 (1.67-3.31) |
| Peru | 0.97 (0.22-1.73) | 4.47 (0.99-8.01) | 5.44 (1.16-9.86) | 15.01 (3.21-27.19) | 3.47 (2.96-3.98) |
| Sudan | 1.32 (0.30-2.33) | 6.60 (1.49-11.65) | 6.36 (1.48-11.59) | 14.64 (3.40-26.69) | 2.67 (1.65-3.70) |
| Indonesia | 8.59 (1.89-15.19) | 4.65 (1.02-8.21) | 39.59 (8.68-68.80) | 14.19 (3.11-24.67) | 2.80 (2.25-3.35) |
| Viet Nam | 3.23 (0.69-5.57) | 4.73 (1.02-8.16) | 13.76 (2.96-24.41) | 13.72 (2.96-24.35) | 2.96 (2.44-3.49) |
| India | 42.42 (9.53-75.85) | 4.97 (1.12-8.89) | 170.21 (37.07-302.32) | 12.03 (2.62-21.37) | 2.15 (1.55-2.76) |
| Maldives | 0.02 (0.00-0.03) | 7.91 (1.75-14.28) | 0.06 (0.01-0.11) | 11.43 (2.50-20.78) | 0.15 (-0.72-1.03) |
| Timor-Leste | 0.03 (0.01-0.05) | 3.85 (0.88-6.83) | 0.15 (0.03-0.28) | 11.04 (2.40-19.93) | 3.07 (2.14-4.02) |
| Yemen | 0.61 (0.14-1.16) | 4.45 (1.01-8.49) | 3.06 (0.72-5.75) | 9.10 (2.14-17.10) | 2.01 (0.75-3.28) |

**NOTE:** ASDR: Age-Standardized Disability Rate; EAPC: Estimated Annual Percentage Change; UI: Uncertainty Interval; CI: Confidence Interval

**Supplementary Table S3**. Interrupted Time Series Analysis of COVID-19 Pandemic Impact on Type 2 Diabetes Mellitus (T2DM) Burden Attributable to High Processed Meat Intake

| **Region** | **ASMR (per 100,000)** | **ASDR (per 100,000)** |  |  |
| --- | --- | --- | --- | --- |
|  | Immediate Effect¹ | Trend Change² | Immediate Effect¹ | Trend Change² |
| Low SDI | 0.036 | -0.021 | 1.009 | -0.050 |
| Low-middle SDI | 0.010 | -0.021 | 0.831 | -0.345 |
| Middle SDI | -0.012 | -0.015 | 1.100 | -0.449 |
| High-middle SDI | 0.120 | -0.024 | 2.442 | -0.476 |
| High SDI | -0.036 | 0.025 | 0.624 | 1.289 |
| Global | 0.023 | -0.005 | 0.966 | -0.046 |

**NOTE:** Immediate Effect: Level change in ASMR/ASDR at the pandemic onset (2020); Trend Change: Slope change in ASMR/ASDR during the 2020–2021 pandemic period; All statistical tests yielded *p*-values > 0.05, indicating no statistically significant effects of the COVID-19 pandemic on T2DM burden attributable to high processed meat intake across regions or globally.; ASMR: Age-Standardized Mortality Rate; ASDR: Age-Standardized Disability Rate
