# Supplementary material for: A systematic analysis of the global disease burden of type 2 diabetes mellitus attributable to high intake of processed meat in 204 countries (1990-2021)
Source: Front Endocrinol (Lausanne). 2025 Sep 30;16:1635831. doi: 10.3389/fendo.2025.1635831 (PMC12518099; doi:10.3389/fendo.2025.1635831)
Supplement: Supplementary file 2 [file DataSheet2.docx]

***Supplementary Figures***

**Supplementary Figure S1** Mechanistic Pathways of Processed Meat-Induced Type 2 Diabetes Conceptual framework illustrating biological mechanisms linking processed meat consumption to T2DM development. Five key components and their specific pathophysiological effects converge through insulin resistance, β-cell dysfunction, and chronic inflammation. Vulnerability factors highlight differential susceptibility across demographic groups.

**Supplementary Figure S2** Quadratic SDI-Disease Burden Relationships

Multivariate quadratic regression analysis showing (A) inverted-U pattern for mortality trends (R² = 0.357, p < 0.001, optimal SDI point = 0.468) and (B) similar pattern for disability trends (R² = 0.296, p = 0.001, optimal SDI point = 0.549). Demonstrates that middle-income development phase represents critical period for diabetes burden escalation, with critical SDI range 0.47-0.55 representing highest-risk development phase.

**Supplementary Figure S3** Box plot comparison of age-standardized mortality rate trends across SDI development quintiles (One-way ANOVA: F = 17.1, p < 0.001). Low-middle SDI countries demonstrate highest burden growth (mean EAPC = 1.47 ± 1.24% per year), while high SDI countries show negative trends (mean EAPC = -0.58 ± 1.52% per year),Diamonds represent group means.

**Supplementary Figure S4** COVID-19 pandemic impact patterns showing mortality-disability paradox across socio-demographic development levels

Scatter plot analysis of immediate COVID-19 pandemic effects (2020 level change) on age-standardized mortality rate (ASMR, x-axis) versus age-standardized disability-adjusted life years (ASDR, y-axis) for type 2 diabetes mellitus burden attributable to high processed meat intake. Each point represents a different socio-demographic index (SDI) quintile group, with colors indicating development levels. The plot is divided into four quadrants by reference lines at null effects (Δ = 0)

**Supplementary Figure S5** COVID-19 pandemic impact on type 2 diabetes mellitus burden trends across socio-demographic index (SDI) regions.

shows the immediate level change effects (β₂) and slope change effects (β₃) for age-standardized mortality rates following the 2020 intervention（A). Displays the corresponding effects for age-standardized disability-adjusted life years rates(B). The heatmap color intensity represents effect size per 100,000 population, with red indicating increases and blue indicating decreases

**Supplementary Figure S6** (A) Age-standardized mortality rate（ASMR） trends by SDI region. (B) Age-standardized disability-adjusted life years rate(ASDR) trends by SDI region. Solid lines represent pre-COVID trends (1990-2019), dashed lines show post-COVID trends (2020-2021). The red vertical line indicates COVID-19 intervention point in 2020. Circles and triangles represent observed data points for pre-COVID and post-COVID periods, respectively.
