# Supplementary figures and images for: A systematic analysis of the global disease burden of type 2 diabetes mellitus attributable to high intake of processed meat in 204 countries (1990-2021)
Source: Front Endocrinol (Lausanne). 2025 Sep 30;16:1635831. doi: 10.3389/fendo.2025.1635831 (PMC12518099; doi:10.3389/fendo.2025.1635831)

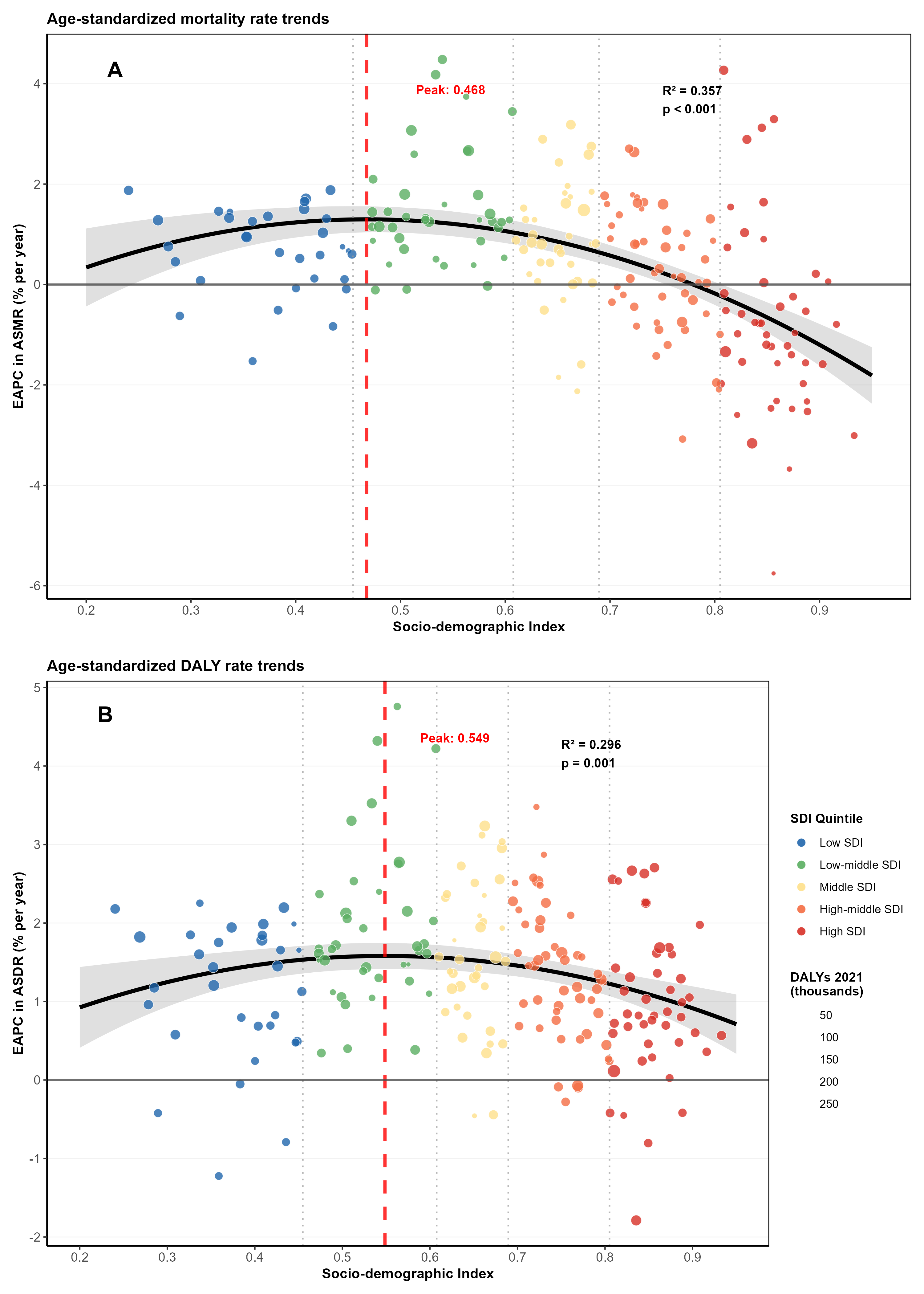

Supplement: Supplementary file 3 [file Image1.tiff]

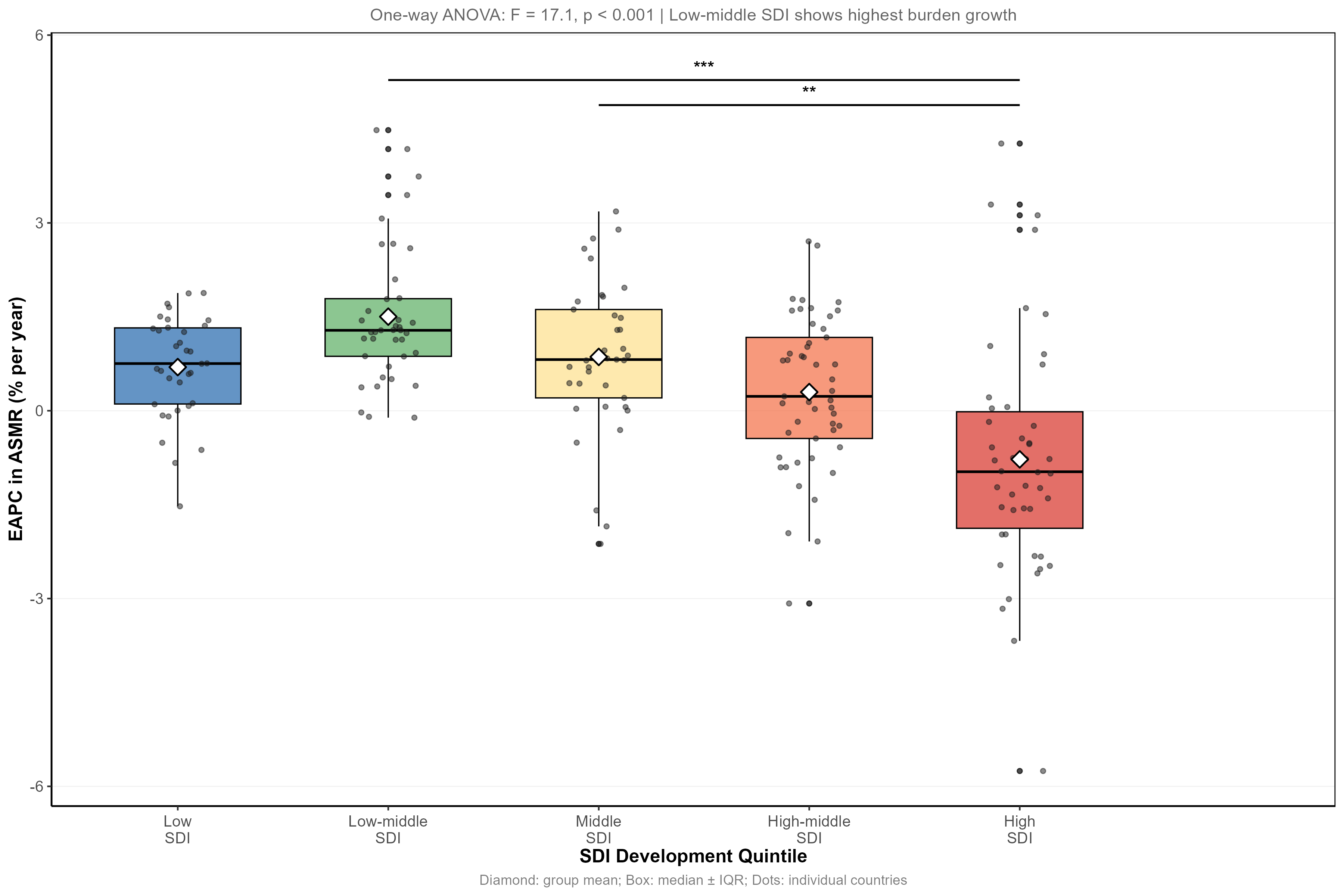

Supplement: Supplementary file 4 [file Image2.tiff]

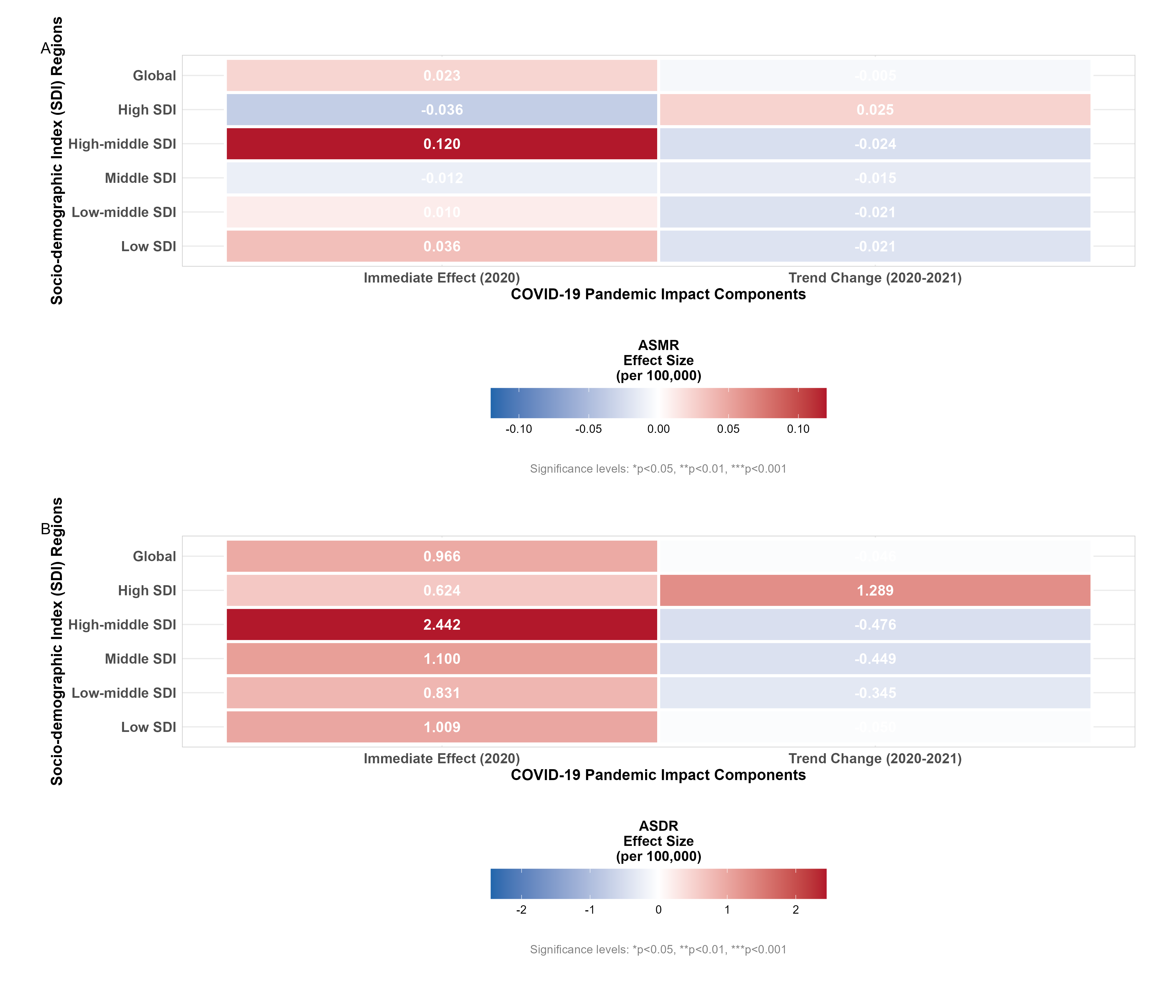

Supplement: Supplementary file 5 [file Image3.tiff]

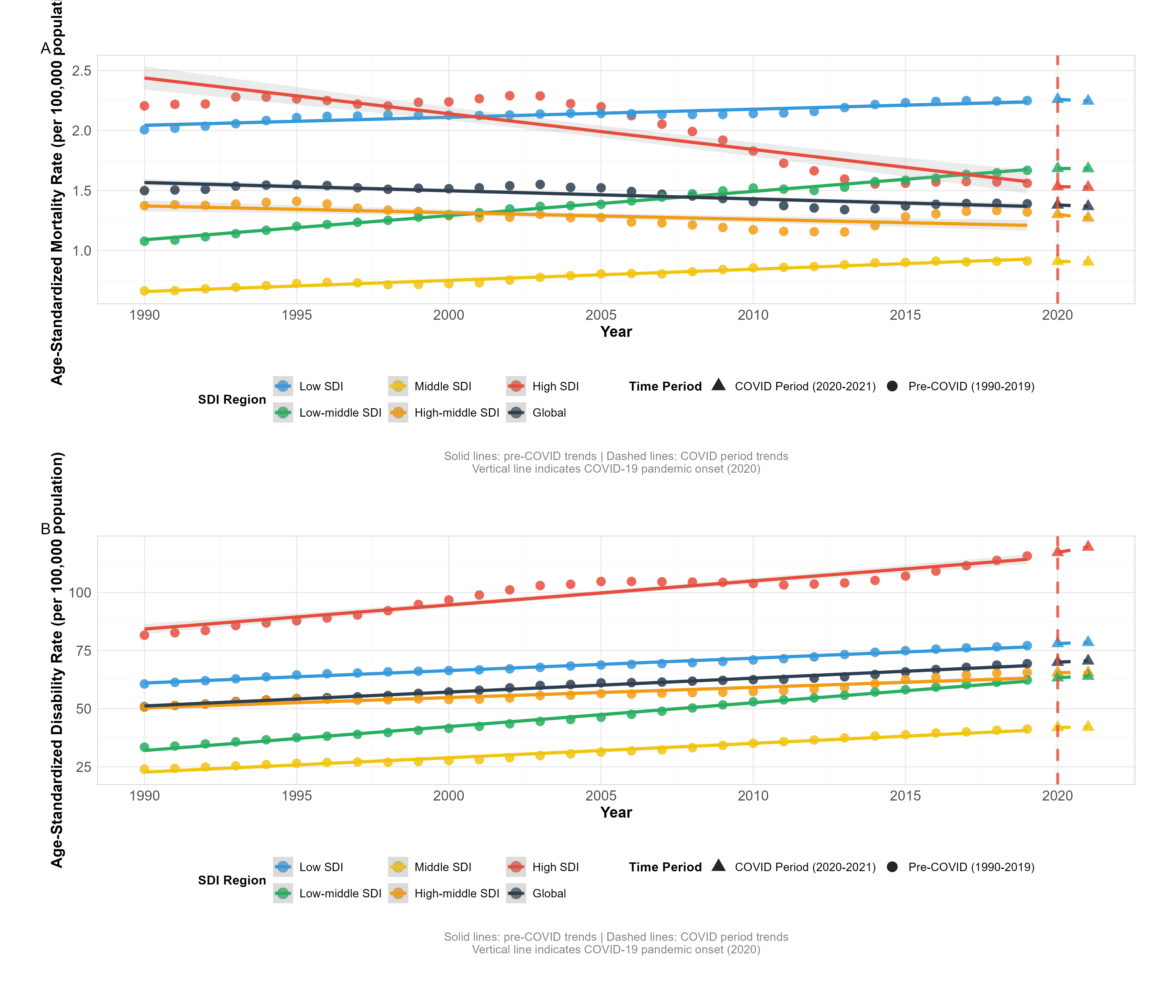

Supplement: Supplementary file 6 [file Image4.tiff]

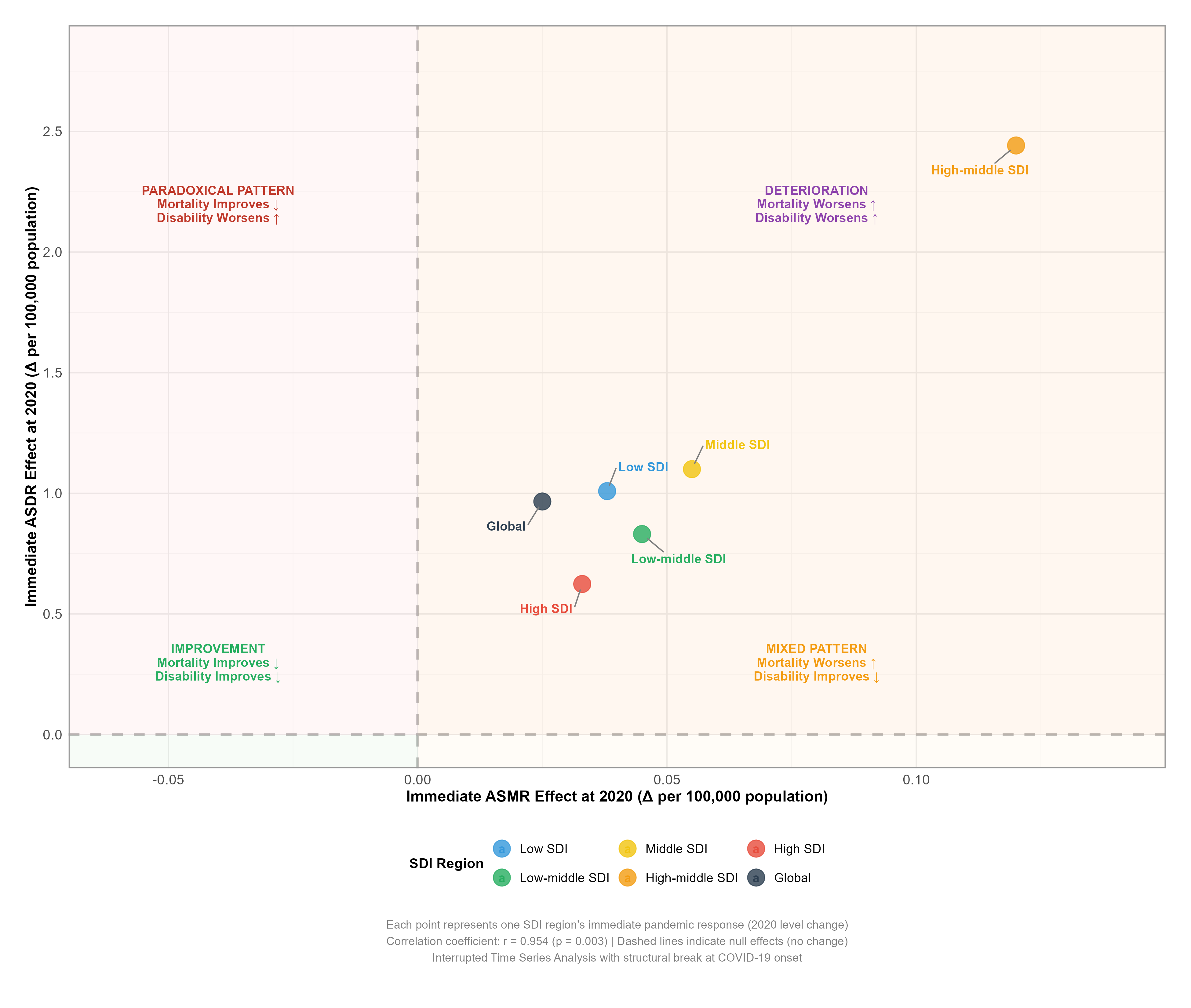

Supplement: Supplementary file 7 [file Image5.tiff]

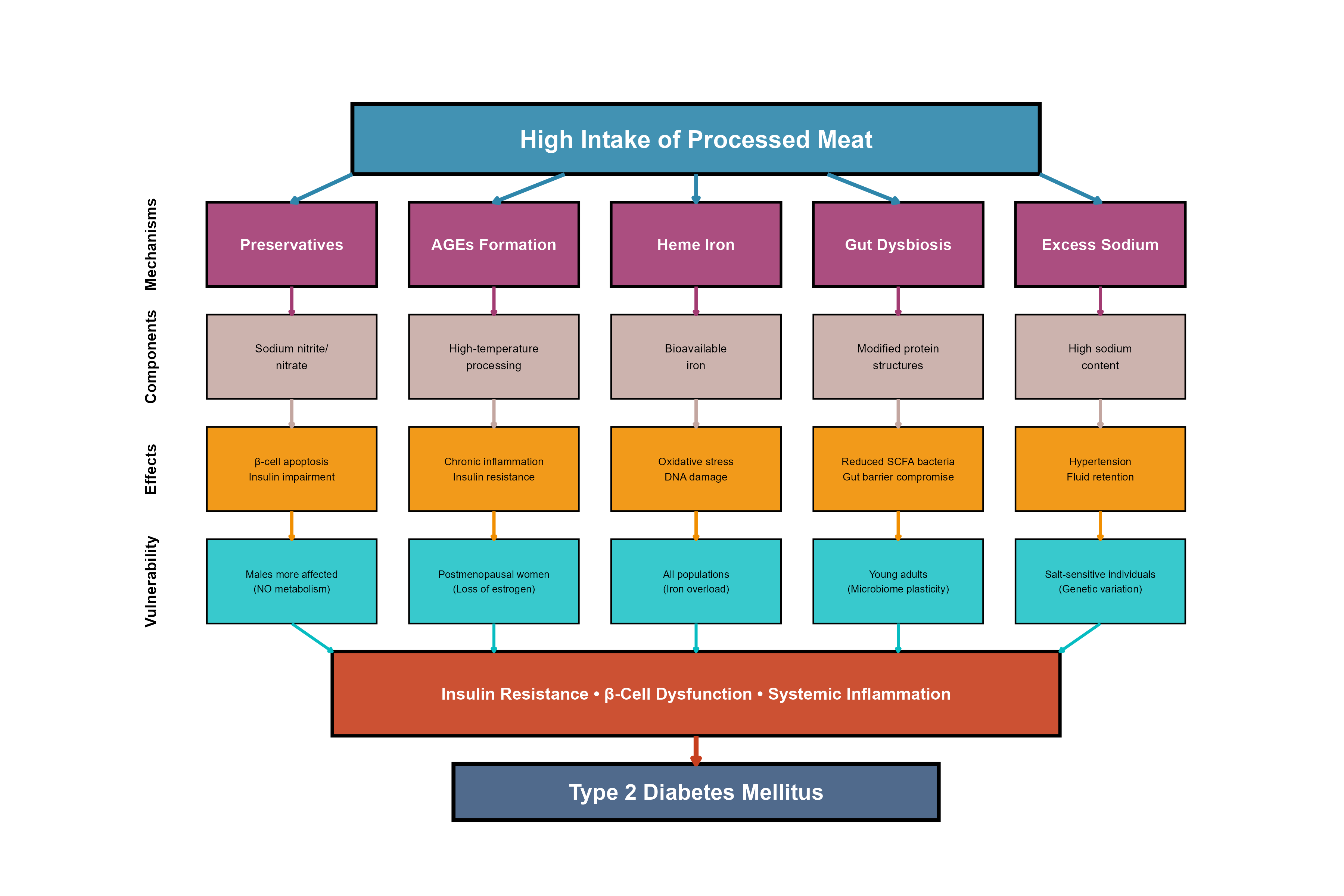

Supplement: Supplementary file 8 [file Image6.tiff]
